# The Mitochondrial Protein RESISTANCE to APHIDS 9 Interacts with S40 to Resist Aphid Infestation by Modulating Reactive Oxygen Species Homeostasis in Maize (*Zea mays*)

**DOI:** 10.1002/advs.202504382

**Published:** 2025-09-24

**Authors:** Chuanhong Wang, Xinqiao Zhang, Zhen Tao, Lei Wang, Shijie Huang, Tengyue Wang, Yibing Zhao, Jinghui Dong, Jing Ma, Chang Chang, Xingzhi Chen, Ning Lin, Peijin Li

**Affiliations:** ^1^ The National Key Engineering Lab of Crop Stress Resistance Breeding the School of Life Sciences Anhui Agricultural University Hefei 230036 China; ^2^ Center for Crop Pest Detection and Control Anhui Agricultural University Hefei 230036 China

**Keywords:** aphids (*Rhopalosiphum maidis*), DUF641, maize (*Zea mays*), mitochondrial protein, reactive oxygen species (ROS) homeostasis, RTA9, S40

## Abstract

As the corn aphid (*Rhopalosiphum maidis*) poses a major threat to maize (*Zea mays*) growth, there is much interest in identifying aphid resistance genes. In this study, an aphid‐susceptible maize mutant from an ethyl methanesulfonate–mutagenized library is identified that exhibits greater aphid settlement than the wild type. Using the MutMap approach, the causal gene *RESISTANCE TO APHIDS 9* (*RTA9*) is cloned, which encodes a mitochondrion‐localized protein from the Domain of Unknown Function 641 family. Overexpressing *RTA9* in maize confers significant resistance to aphids without compromising seed yield. It further identifies the senescence regulator *S40* as an interactor of *RTA9*, which negatively regulates the stability of *S40*. Knockout of *S40* enhanced aphid resistance, while its overexpression increased susceptibility. Further analysis demonstrates that the *rta9‐1* mutant does not exhibit significant enrichment of differentially expressed genes associated with oxidoreductase activity following aphid infestation. By contrast, genes involved in this pathway are significantly enriched in the *s40* mutant. Additionally, aphid‐induced reactive oxygen species (ROS) levels are markedly lower in *rta9‐1* than in the wild type but significantly higher in *s40*. Collectively, the results suggest that the mitochondrial protein *RTA9* and its interacting partner *S40* regulate resistance to aphid infestation by modulating ROS homeostasis.

## Graphical abstract

This study identifies the mitochondrial protein *RTA9*, a member of the Domain of Unknown Function 641 family, together with its partner *S40*, as key regulators of aphid resistance in maize. *RTA9* promotes *S40* degradation and modulates reactive oxygen species accumulation. Either overexpression of *RTA9* or knockout of *S40* enhances aphid resistance without yield penalties, offering promising genetic targets for developing aphid‐resistant crops.

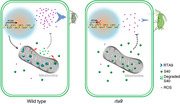

## 1 Introduction

Aphids (Hemiptera: Aphididae) are prominent and destructive pests in crop production that obtain nutrients from their host by piercing the plant tissues and sucking the phloem sap.^[^ ^1^ ^]^ Due to their rapid reproduction cycle, aphid infestations can spread quickly, severely compromising crop nutrient acquisition and suppressing plant growth.^[^ ^2^ ^]^ Aphids also secrete honeydew, which can adhere to the surface of leaves and, in maize (*Zea mays*), to the tassels, resulting in lower photosynthetic rate and pollination efficiency, respectively, and negatively affecting crop yield and quality.^[^ ^3^, ^4^ ^]^ Aphids are also vectors for certain viruses and can transmit diseases that reduce yield or even lead to plant death.^[^ ^5^, ^6^, ^7^ ^]^

Maize is an important cereal crop worldwide, serving as a staple source of human food, animal feed, and industrial materials.^[^ ^8^, ^9^ ^]^ The corn aphid *Rhopalosiphum maidis* has become a major cause of yield loss in maize production.^[^ ^10^ ^]^ In agriculture, breeding aphid‐resistant varieties is the most ecologically‐friendly and effective approach to alleviating the threats posed by this pest.^[^ ^11^ ^]^ Extensive efforts have been devoted to dissecting the genetic basis and molecular mechanisms of aphid resistance in maize, yet due to the complexity of aphid populations, their diverse hosts and the influences of local environmental factors in the field, progress in aphid control research has been limited.

Plants are constantly evolving physical and chemical barriers that prevent insects from successfully colonizing and feeding on their tissues.^[^ ^1^, ^12^, ^13^ ^]^ Epicuticular waxes and glandular trichomes provide a physical barrier against non‐adapted aphids,^[^ ^14^ ^]^ while several plant chemical compounds have anti‐nutritive, repellent, or toxic effects on aphids, such as callose,^[^ ^15^ ^]^ betulin,^[^ ^16^ ^]^ flavonoids, and free gossypol.^[^ ^17^ ^]^ Such plant substances have been the focus of research aimed at controlling aphids through multiple approaches.^[^ ^18^, ^19^ ^]^ For instance, the ectopic expression of an agglutinin gene from the traditional Chinese herb crow‐dipper (*Pinellia ternata*) in tobacco (*Nicotiana tabacum*) conferred broad‐spectrum resistance to aphids and other insects.^[^ ^19^ ^]^

At the molecular level, the signaling mechanisms of anti‐insect pathways in plants are important for both local immune responses and the propagation to systemic tissues, including the propagation of electrical signals, calcium influx, reactive oxygen species (ROS) production, and the plant hormones, such as salicylic acid (SA) and jasmonic acid (JA).^[^ ^20^ ^]^ Among these pathways and molecules, ROS act as a shield against insect invasion and serve as a central regulator of various biological programs, allowing cells to respond rapidly to different stimuli.^[^ ^21^ ^]^ Elevated cellular ROS levels, known as a ROS burst, enhance the plant's resistance by activating the expression of defense‐related genes,^[^ ^22^, ^23^ ^]^ reprogramming signaling networks, and modifying plant secondary metabolic pathways to reduce insect feeding, growth, and survival.^[^ ^21^, ^24^ ^]^ Conversely, the inhibition of ROS bursts promotes aphid colonization in the host plant.^[^ ^22^, ^25^ ^]^ For instance, lower ascorbate oxidase activity results in a more pronounced reduction of the apoplastic redox state, compromising host resistance to aphids. This resistance manifests through multifaceted changes, including altered cell wall composition, transcriptional reprogramming of phytohormone‐mediated defense genes, and metabolic shifts in amino acids and sugars.^[^ ^26^ ^]^ In Arabidopsis (*Arabidopsis thaliana*), plants harboring a null mutation for *RBOHD*, which encodes the apoplastic NADPH oxidase RESPIRATORY BURST OXIDASE HOMOLOG D, support accelerated colony expansion of the green peach aphid (*Myzus persicae*) compared to their wild‐type counterparts, indicating that ROS generation is essential for mounting fully effective defense responses.^[^ ^27^ ^]^ In wheat (*Triticum aestivum*), H_2_O_2_ accumulation inhibits the proliferation of apterous (wingless) aphids while promoting the production of alate (winged) aphids.^[^ ^28^ ^]^ Through evolutionary adaptation, aphids have developed countermeasures against host defenses: the salivary effector proteins Mp55 from *M. persicae* and Histidine‐rich calcium‐binding protein (ApHRC) from the pea aphid (*Acyrthosiphon pisum*) inhibit host ROS production, significantly enhancing aphid fecundity.^[^ ^25^, ^29^ ^]^ The salivary protein Cathepsin B3 (CathB3), a cysteine protease, suppresses feeding by triggering phloem‐localized ROS accumulation; thus, aphids that express high levels of *CathB3* exhibit lower colonization success due to feeding‐induced ROS bursts, while aphids with low *CathB3* expression have enhanced colonization fitness.^[^ ^22^ ^]^

Mitochondria are the main organelles responsible for utilizing and converting chemical energy into a usable form, which requires the activity of the many core subunits of the electron transport chain (ETC). Mitochondria are a major source of ROS in plant cells.^[^ ^30^ ^]^ Recent studies have shown that several mitochondrion‐localized proteins participate in aspects of plant biology beyond energy production or conversion, such as thermotolerance, kernel development, leaf senescence, and disease resistance.^[^ ^31^, ^32^, ^33^, ^34^, ^35^ ^]^ Those mitochondrion‐localized proteins involved in plant tolerance or resistance are typically associated with ROS pathways; for example, the mitochondrial RNA processing factor RESISTANCE TO PHYTOPHTHORA PARASITICA 7 (RTP7) mediates plant immunity to a broad spectrum of pathogens by modulating mitochondrial ROS accumulation.^[^ ^34^ ^]^ The roles and mechanisms of mitochondrial proteins in aphid resistance are unclear in crops; however, understanding how these proteins function in this context is essential for devising strategies to enhance aphid resistance during maize breeding.

Domain of Unknown Function 641 (DUF641) is a domain found in members of a plant‐specific protein family, but its functions are unclear. While current evidence implicates DUF641‐containing proteins in regulating organ size,^[^ ^36^ ^]^ light‐dependent hypocotyl elongation,^[^ ^37^ ^]^ and drought tolerance or water usage,^[^ ^38^, ^39^ ^]^ no association has been documented with aphid resistance to date. Here, we identified an aphid‐susceptible maize mutant and cloned the causal gene, *RESISTANCE TO APHIDS 9* (*RTA9*; Zm00001d039444), which encodes a mitochondrion‐localized protein containing a DUF641 domain. Loss of *RTA9* function impairs the ROS signaling response to aphid stimulation in maize, leading to imbalanced ROS levels. Overexpression of *RTA9* conferred greater aphid resistance without negatively affecting grain yield. Further investigations revealed that *RTA9* interacts with *S40*, a factor that regulates senescence, and affects its protein stability. Maize plants that overexpressed *S40* had reduced ROS levels and attenuated aphid resistance, whereas the *s40* mutant exhibited the opposite phenotype. These observations suggest that the maize mitochondrial protein *RTA9* responds to aphid infestation by mediating ROS production signals, making it a potential candidate gene for developing new strategies to manage aphids in maize.

## 2 Results

### 2.1 The Maize Mutant *rta9‐1* is Susceptible to Aphids

We screened an ethyl methanesulfonate (EMS)‐induced mutant maize population for aphid resistance and identified an aphid‐susceptible plant, which we designated *resistance to aphids 9‐1* (*rta9‐1*). Many aphids colonized the sheaths and leaves of mature *rta9‐1* seedlings; the susceptible plants had ≈ 10 times more aphids than the wild type, the maize inbred line B73 (Figure S1, Supporting Information).

**Figure 1:**
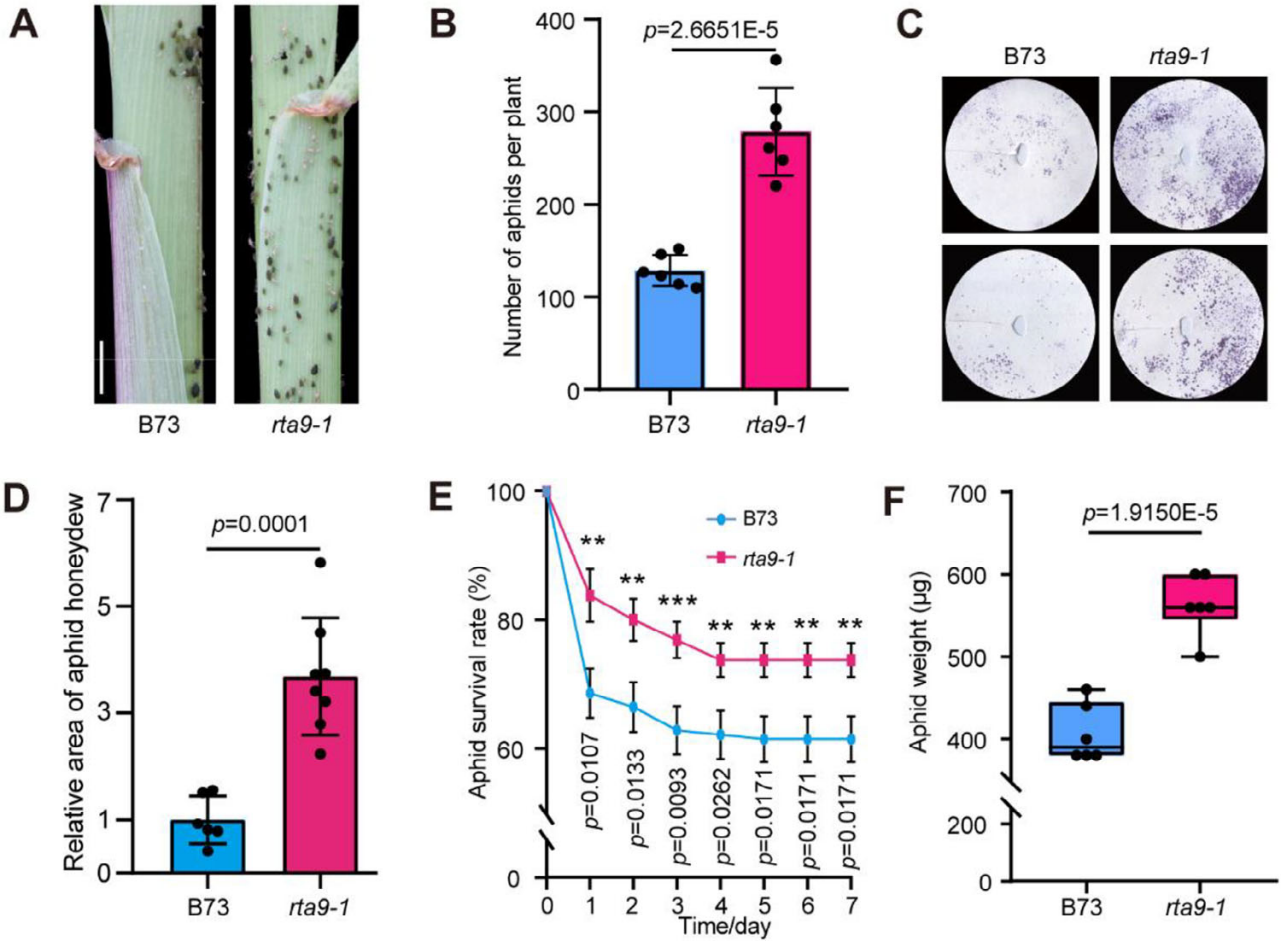
The *rta9‐1* mutant is susceptible to aphids. A) Colonization and distribution of aphids on the wild‐type B73 and the *rta9‐1* mutant after 21 days of infestation. Scale bar, 1 cm. B) Quantification of aphids growing on B73 and *rta9‐1* as shown in (A). C,D) Honeydew secretion assay of aphid feeding. The quantification of the honeydew areas on the filter papers in (C) is shown in (D). E) Survival rates of aphids feeding on B73 or *rta9‐1* (^**^ *p* < 0.01, ^***^ *p* < 0.001). F) Body weight of each aphid feeding on B73 or *rta9‐1*. The boxes represent the interquartile range, with the middle line defining the median. The lines extending from the quartiles of the box are called “whiskers” and show the maximum and minimum values. In panels (B, D–F), at least six independent replicates were performed for each assay, and the data in panels (B, D–E) are presented as means ± standard deviation (SD). The values in (D) were normalized to B73, which was set to 1. Statistical significance of the differences between B73 and *rta9‐1* was determined by Student's *t*‐tests.

To rule out the possibility that this phenotype might be caused by chance or environmental bias, we self‐crossed *rta9‐1* and repeated the aphid bioassays on the progeny of *rta9‐1* under controlled greenhouse conditions. As observed for the parental seedlings grown in the field, many aphids colonized the sheaths of *rta9‐1* progeny after 21 days of infestation when grown under control conditions (**Figure** **1**A,B). In addition, significantly more honeydew (purple‐red area on the filter paper; Figure 1C,D) was secreted by aphids feeding on *rta9‐1* progeny plants than by those feeding on B73. After one week of infestation, the aphid survival rates were significantly higher on the *rta9‐1* progeny seedlings than on B73 (Figure 1E). Furthermore, the aphids feeding on the *rta9‐1* progeny weighed more than those feeding on the wild type (Figure 1F). Therefore, *rta9‐1* appears to be an aphid‐susceptible mutant that is more suitable for aphid colonization.

### 2.2 Cloning *RTA9* using the Mutmap Approach

To clone *RTA9*, we backcrossed the aphid‐susceptible mutant *rta9‐1* with the wild‐type B73 to obtain *rta9‐1/RTA9* F_1_ seeds. The F_1_ plants exhibited the same levels of aphid resistance as B73, with similar aphid survival rates and honeydew secretion levels, but significantly lower levels than *rta9‐1* (Figure S2A–C, Supporting Information). Therefore, the aphid‐susceptibility phenotype of *rta9‐1* may be caused by a recessive mutation.

**Figure 2:**
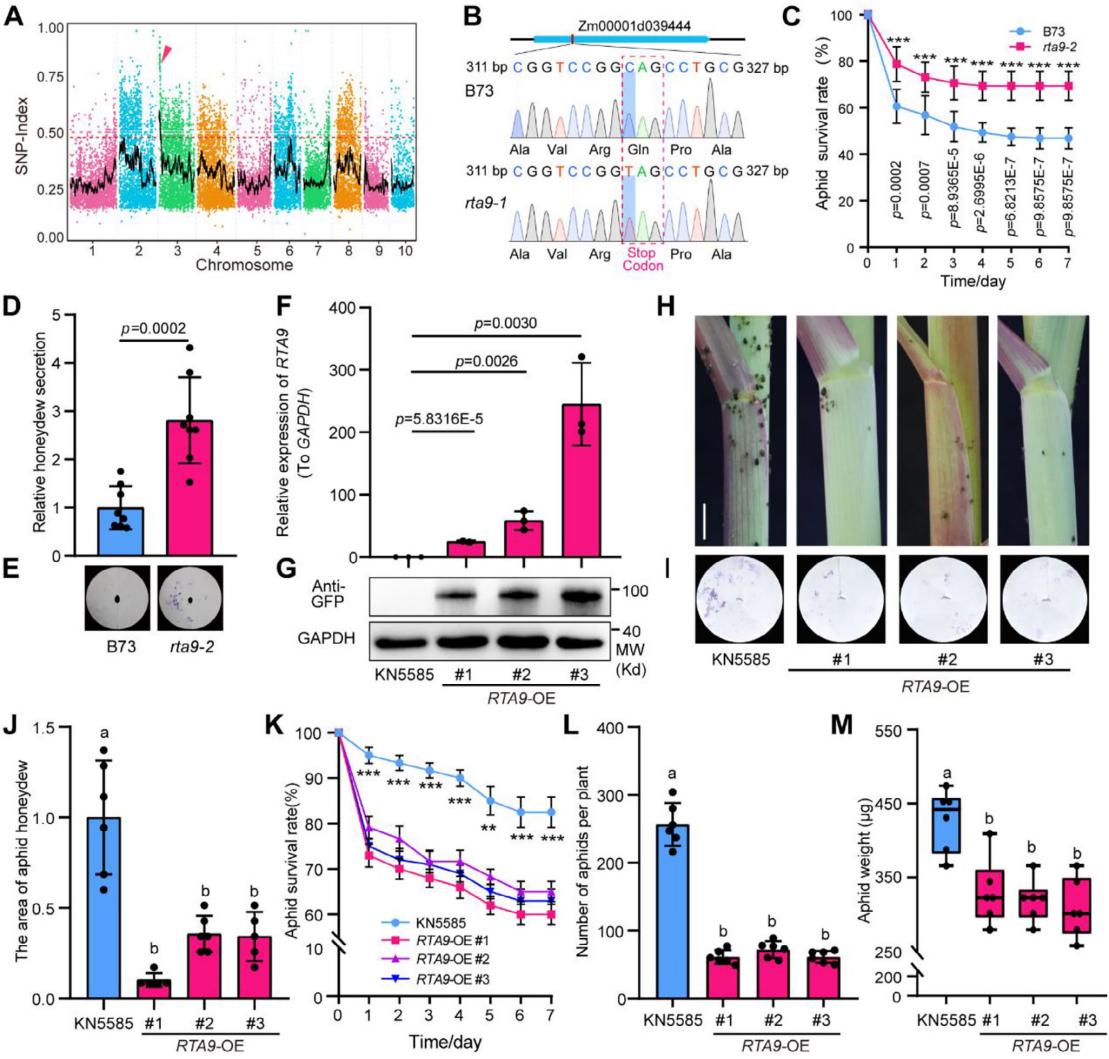
MutMap‐based cloning of *RTA9* and aphid resistance tests of the *rta9‐2* mutant and *RTA9‐*overexpressing plants. A) Distribution of CG>TA‐type mutations in *rta9‐1* across the 10 maize chromosomes, shown as the SNP‐index. The red arrowhead marks the peak over the significance threshold on chromosome 3. B) Diagram of the Zm00001d039444 (*RTA9*) locus and confirmation of the C‐to‐T mutation in *rta9‐1* by Sanger sequencing. The red box shows the codon change from CAG to TAG, a stop codon that is predicted to result in a truncated *RTA9* protein. C) Survival rate of aphids feeding on B73 or the independent mutant allele *rta9‐2* (^***^ *p* < 0.001). D,E) Honeydew secretion assay. Quantification of the honeydew areas on the filter paper in (E) is presented in (D). F) Relative *RTA9* transcript levels in the wild‐type KN5585 and *RTA9‐*overexpressing (*RTA9‐*OE) transgenic lines. *GAPDH* was used as an internal reference for the RT‐qPCR analysis. G) Immunoblot analysis indicating that *RTA9‐GFP* accumulates in the *RTA9‐*OE transgenic plants. GAPDH was used as a loading control. H) Colonization and distribution of aphids on the wild‐type KN5585 and *RTA9‐*OE lines after 21 days of infestation. Scale bar, 1 cm. I,J) Honeydew secretion assay. Quantification of the honeydew areas on the filter paper in (I) is shown in (J). K) Survival rate of aphids feeding on KN5585 or *RTA9‐*OE lines (^**^ *p* < 0.01, ^***^ *p* < 0.001). L,M) Number and body weight of colonizing aphids after 21 days of infestation. In (F), data are shown as means ± SD, *n* = 3. In (C, D, J–L), the data are shown as means ± SD, *n* = 6. In (M), the boxes represent the interquartile range, with the middle line defining the median. The lines extending from the quartiles of the box are called “whiskers” and show the maximum and minimum values. Statistical significance was determined using Student's *t*‐tests (C, D, F, K) or one‐way ANOVA followed by Tukey's test (J–M) (*p* < 0.05), with different letters indicating significant difference.

We followed the MutMap approach^[^ ^40^ ^]^ to identify *RTA9*. To this end, we selected 25 aphid‐susceptible seedlings from the segregating F_2_ population derived from the *rta9‐1/RTA9* F_1_ plants, extracted their genomic DNA, and pooled equal amounts of the DNA for library construction and genome sequencing. We identified 17809 single‐nucleotide polymorphisms (SNPs) in the bulk sequencing data, and SNP‐index analysis revealed a major peak containing 384 closely linked SNPs on chromosome 3 (**Figure** **2**A). Of these SNPs in the candidate region, most were located in the intergenic (299), upstream (34), downstream (27), and intronic (13) regions of genes. Within the exon regions, a C‐to‐T substitution caught our attention at position 4498925, resulting in the conversion of Gln (CAG) to a stop codon (TAG). This change is predicted to lead to the premature termination of translation of the protein encoded by Zm00001d039444 (Figure 2B). We designed a derived cleaved amplified polymorphic sequence (dCAPS) marker and sequenced PCR products spanning this substitution using Sanger sequencing to assess mixed DNA pools of aphid‐susceptible and aphid‐resistant individuals from another F_2_ population. The pool with the mutant phenotype was homozygous for this C‐to‐T SNP, while the other DNA pools exhibited either one wild‐type sequencing peak or a mix of two peaks at this position (Figure S3, Supporting Information).

To test whether the putative C‐to‐T mutation in Zm00001d039444 was responsible for the aphid susceptibility of *rta9‐1*, we ordered a Mu‐insertion mutant designed *rta9‐2* from the ChinaMu database^[^ ^41^ ^]^ (Figure S4, Supporting Information). Indeed, this Mu‐insertion mutant exhibited a similar aphid susceptibility phenotype as the EMS mutant *rta9‐1* (Figure 2C–E). Therefore, Zm00001d039444 appears to be the causal gene for the aphid‐susceptible phenotype observed in *rta9* mutants. We hereafter refer to Zm00001d039444 as *RTA9*.

*RTA9* is a single‐exon gene that encodes a plant‐specific Domain of Unknown Function 641 (DUF641) protein and has not been previously reported to be associated with aphid resistance. We detected transcripts for *RTA9* in various B73 tissues. Relatively high expression levels were observed in the tassel, followed by the ear, with the lowest levels in the leaf (Figure S5A, Supporting Information). In addition, we examined *RTA9* transcript levels at different time points following aphid infestation. The results showed that *RTA9* expression initially exhibited a slight increase, followed by a transient decrease, and then peaked at 24 h post‐infestation (Figure S5B, Supporting Information).

### 2.3 Overexpression of *RTA9* in Maize Confers Aphid Resistance without Negatively Affecting Grain Yield

To validate the role of *RTA9* in aphid resistance, we generated transgenic lines that overexpress *RTA9* (*RTA9‐*OE) in the KN5585 background, with the sequence encoding green fluorescent protein (GFP) cloned in‐frame and downstream of *RTA9*. We validated the transgenic lines using reverse‐transcription quantitative PCR (RT‐qPCR), which revealed that *RTA9* transcript levels were significantly upregulated in the *RTA9*‐OE lines relative to the wild type (Figure 2F). In addition, an immunoblot analysis using anti‐GFP antibodies indicated that *RTA9‐GFP* accumulates in these transgenic plants (Figure 2G).

To evaluate the effect of *RTA9* overexpression on aphids, we counted the number of aphids on transgenic plants. The *RTA9*‐OE lines had significantly fewer aphids than the wild‐type seedlings after 21 days of aphid infestation. Consistent with this result, the amount of excreted honeydew was much lower on the *RTA9*‐OE seedlings than on the wild‐type seedlings (Figure 2H–K). The *RTA9*‐OE plants had only ≈ 25% the number of aphids present on the wild type, and the aphids colonizing the *RTA9*‐OE plants had a lower average weight than those colonizing the wild type (Figure 2L,M). In many cases, resistance genes that enhance plant defense come with trade‐off effects.^[^ ^42^ ^]^ Therefore, we examined the agronomic and yield‐related traits of *RTA9*‐OE lines and the wild‐type KN5585. *RTA9*‐OE plants exhibited reduced plant height and ear height compared to KN5585, but had longer and wider ear leaves (Figure S6A, Supporting Information). Importantly, overexpression of *RTA9* did not negatively impact seed production, including traits such as ear weight, hundred‐kernel weight, and grain weight per ear, under field conditions. (Figure S6B,C, Supporting Information). Taken together, we conclude that *RTA9* overexpression improves aphid resistance in maize without negatively affecting grain yield.

### 2.4 *RTA9* Physically Interacts with *S40*

To dissect the mechanism of *RTA9* function, we analyzed the protein–protein interaction network of *RTA9* using the STRING online tool (https://cn.string‐db.org/cgi/input?sessionId = bgtEzKim8hnP&input_page_show_search = on). We identified the senescence‐regulating protein *S40*, encoded by Zm00001d003009, as a potential interaction partner of *RTA9*.

**Figure 3:**
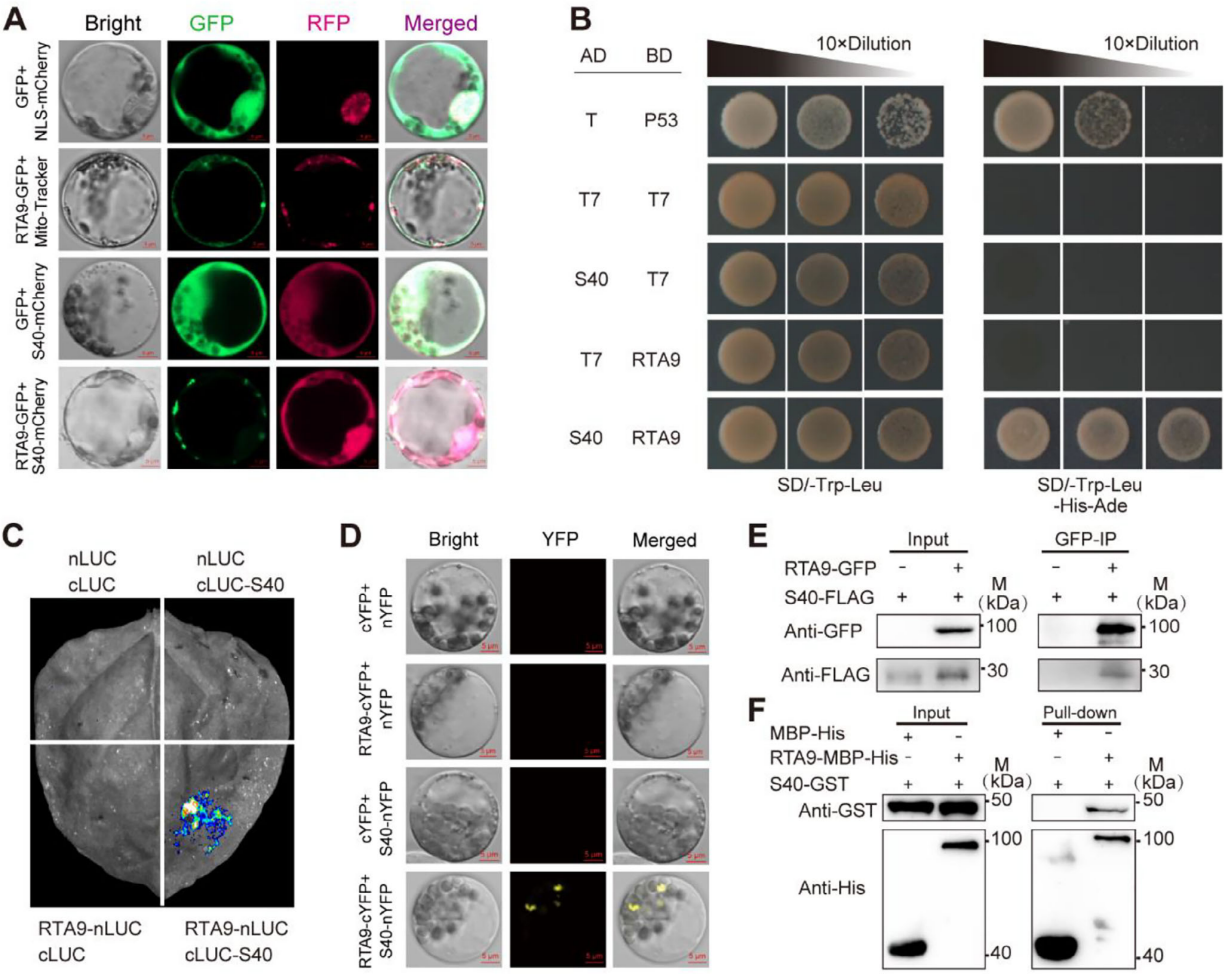
*RTA9* interacts with *S40* in vivo and in vitro. A) Co‐localization of *RTA9* and *S40* in maize protoplasts. Mitochondria were visualized by staining with MitoTracker Red dye. Nuclei were visualized with the nuclear marker NLS‐mCherry. Free GFP was used as a control for *RTA9‐GFP*. B) Yeast two‐hybrid assay indicating that *RTA9* interacts with *S40* in yeast cells. Positive colonies were spotted onto synthetic defined (SD) medium lacking Trp and Leu (SD/−Trp−Leu) and SD medium lacking Trp, Leu, His, and Ade (SD/−Trp−Leu−His−Ade). The T + P53 combination was used as a positive control. C) Firefly luciferase (LUC) complementation imaging assay indicating that *RTA9* interacts with *S40* in the leaves of *N. benthamiana* plants. D) Bimolecular fluorescence complementation (BiFC) assay showing that *RTA9* interacts with *S40* in maize protoplasts. E) Co‐immunoprecipitation (Co‐IP) assay demonstrating the interaction between *RTA9‐GFP* and *S40‐FLAG* in vivo. The *RTA9‐GFP* transgenic line was crossed with the *S40‐FLAG* transgenic line, and the derived F_1_ generation plants were used for Co‐IP analysis. F) His protein pull‐down assay revealing that RTA9‐MBP‐His interacts directly with *S40‐GST* in vitro.

To test the relationship between *RTA9* and *S40*, we assessed their subcellular localization by transfecting maize protoplasts with a construct encoding *RTA9* fused to GFP (*RTA9‐GFP*) or *S40* fused to the red fluorescent protein mCherry (*S40‐mCherry*). We detected a dotted cellular distribution of *RTA9‐GFP* in cells that overlapped with the mitochondrial marker dye Mito Tracker Red, indicating that *RTA9* localizes to mitochondria (**Figure** **3**A; Figure S7, Supporting Information). By contrast, *S40* was distributed throughout the cell, and was present in both the nucleus and the cytosol. The *RTA9‐GFP* signal partially overlapped with that of *S40‐mCherry* (Figure 3A), suggesting that *RTA9* and *S40* are partially co‐localized in mitochondria.

We conducted a targeted yeast two‐hybrid (Y2H) assay to validate the interaction between *RTA9* and *S40*. Yeast cells harboring *pGADT7‐S40* and *pGBKT7‐RTA9* were able to grow on synthetic defined (SD) selective medium (lacking Trp, Leu, His, and Ade), while cells containing all other plasmid combinations did not. This indicates that these two proteins can interact in yeast (Figure 3B). To establish whether this interaction also occurs in plant cells, we used a firefly luciferase (LUC) complementation imaging assay in the leaves of *N. benthamiana* plants. Leaves co‐infiltrated with *nLUC‐RTA9* (encoding the N‐terminal half of LUC fused to *RTA9*) and *cLUC‐S40* (encoding the C‐terminal half of LUC fused to *S40*) exhibited strong LUC activity, whereas the other combinations of constructs did not display any LUC signal (Figure 3C). A bimolecular fluorescence complementation (BiFC) assay confirmed that *RTA9‐cYFP* (a fusion between *RTA9* and the N‐terminal half of yellow fluorescent protein [YFP]) interacts with *S40‐nYFP* (a fusion between *S40* and the C‐terminal half of YFP) when their encoding plasmids are co‐transfected into maize protoplasts (Figure 3D). We also conducted in vivo protein co‐immunoprecipitation (Co‐IP) assays using F_1_ plants harboring the *RTA9‐GFP* and *S40‐FLAG* transgenes. Following immunoprecipitation with an anti‐GFP antibody, we detected *S40‐FLAG* among the precipitated proteins, confirming that *RTA9‐GFP* interacts with *S40‐FLAG* in plant cells (Figure 3E). Finally, a pull‐down assay confirmed that *RTA9* and *S40* interact directly in vitro, as RTA9‐MBP‐His (a fusion of *RTA9* and the maltose‐binding protein [MBP]) was pulled down by S40‐GST (a fusion of *S40* and glutathione S‐transferase [GST]) (Figure 3F). Together, these results indicate that *RTA9* can physically interact with *S40* in vitro and in vivo.

### 2.5 Knockout and Overexpression of *S40* Confirm Its Role in Maize Resistance to Aphids

**Figure 4:**
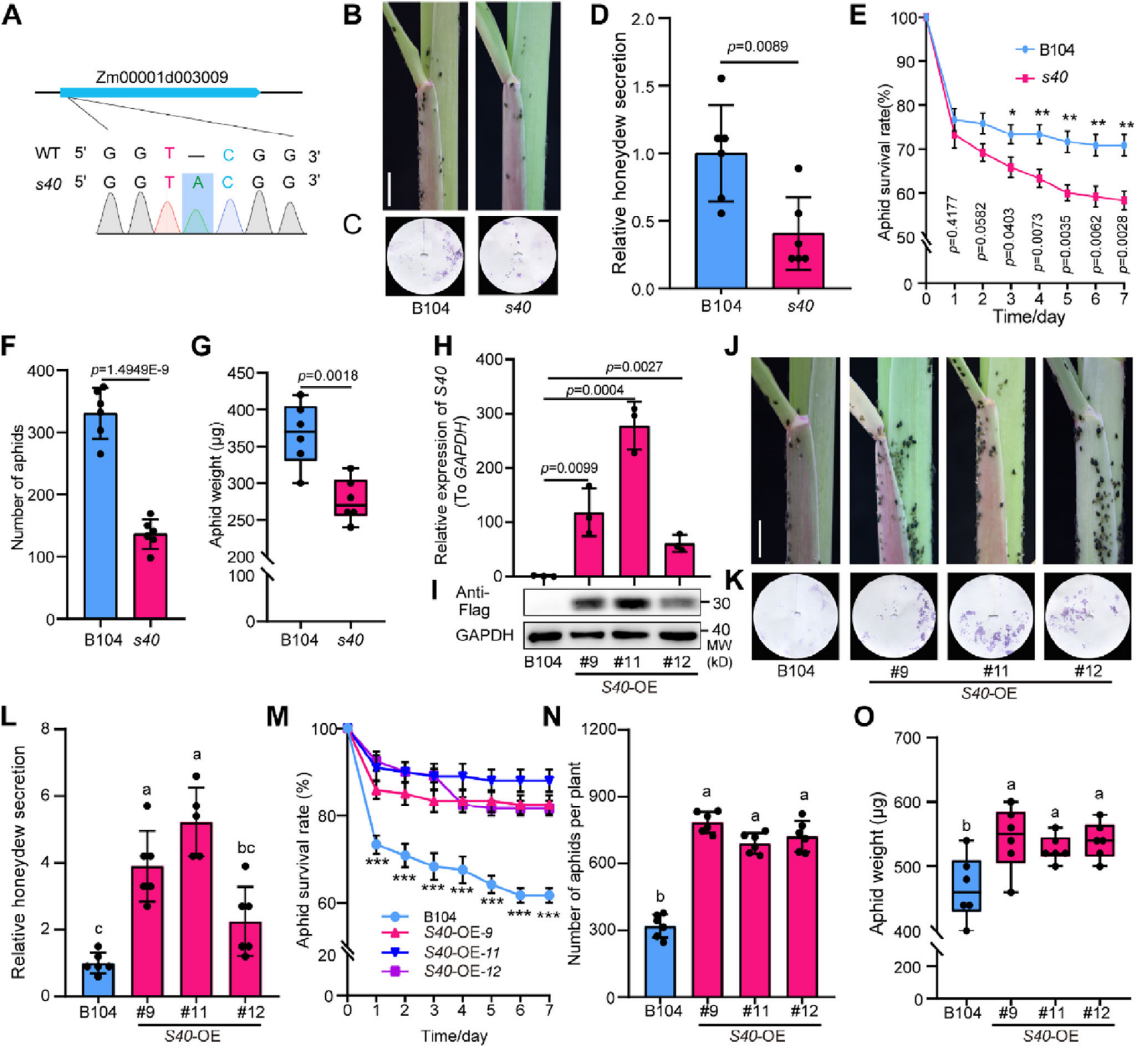
Loss of *S40* function confers aphid resistance, while *S40* overexpression increases aphid susceptibility in maize. A) Diagram of the Zm00001d003009 (*S40*) locus and identification of the mutation in *s40* by Sanger sequencing. B) Colonization and distribution of aphids after 21 days of infestation of the wild‐type B104 and *s40* plants. Scale bar, 1 cm. C,D) Honeydew secretion assay of aphid feeding. Quantification of the honeydew areas on the filter paper in (C) is shown in (D). E) Survival rate of aphids feeding on the wild‐type B104 or *s40* mutant (^*^ *p* < 0.05, ^**^ *p* < 0.01). F) Number of aphids present after 21 days of infestation. (G) Body weight of aphids. H) Relative *S40* transcript levels in B104 and *S40*‐overexpression (*S40*‐OE) transgenic plants. *GAPDH* was used as an internal reference. I) Immunoblot analysis indicating that *S40‐FLAG* accumulates in *S40*‐OE transgenic plants. GAPDH was used as a loading control. J) Colonization and distribution of aphids after 21 days of infestation. Scale bar, 1 cm. K,L) Honeydew secretion assay of aphid feeding. Quantification of the honeydew areas on the filter paper in (K) is shown in (L). M) Survival rate of aphids (^***^ *p* < 0.001). N,O) Number (N) and body weight (O) of colonizing aphids after 21 days of infestation. In (H), data are shown as means ± SD, *n* = 3. In (D–F, I–N), data are shown as means ± SD, *n* = 6. In (G, O), the boxes represent the interquartile range, with the middle line defining the median. The lines extending from the quartiles of the box are called “whiskers” and show the maximum and minimum values. Statistical significance was determined using Student's *t*‐test (D–H) or one‐way ANOVA followed by Tukey's test (L–O) (*p* < 0.05), with different letters indicating significant differences.

*S40* is a single‐exon gene. RT‐qPCR analysis detected *S40* transcripts in various maize tissues, with a relatively high expression level in roots and tassels (Figure S8A, Supporting Information). Upon aphid infestation, *S40* expression exhibited an early decline, peaked at 6 h post‐infestation, and then progressively decreased, reaching its lowest level at 24 h (Figure S8B, Supporting Information). To investigate the possible anti‐aphid function of *S40*, we generated knockout mutants of *S40* in the B104 background using clustered regularly interspaced short palindromic repeats (CRISPR) and CRISPR‐associated nuclease 9 (Cas9)‐mediated gene editing. We verified the identity of one homozygous *s40* knockout mutant by PCR and Sanger sequencing. The *s40* mutant carries a 1‐bp insertion in the *S40* coding region leading to a frameshift in the open reading frame (**Figure** **4**A). Aphid bioassays showed that the loss of *S40* function significantly enhances maize resistance to aphids; indeed, the number of aphids, the amount of honeydew secretion, survival rates, and body weight were significantly lower for aphids feeding on *s40* seedlings than for those feeding on the wild‐type B104 (Figure 4B–G). Similar to the *RTA9*‐OE lines, we evaluated the agronomic and yield‐related traits of the *s40* mutant. The mutant plants showed no significant adverse effects on key agronomic traits, including plant architecture (plant height and ear height), leaf morphology (ear leaf length and width), and seed production (ear length, ear width, ear weight, hundred‐kernel weight, and grain weight per ear) (Figure S9A–C, Supporting Information).

We generated transgenic lines that overexpress *S40‐FLAG* (*S40*‐OE) driven by the *Ubiquitin* promoter in the B104 background and confirmed the presence and expression of the transgenes by PCR and RT‐qPCR, respectively. The transgenic plants had a significantly higher *S40* expression level than the wild type (Figure 4H) and accumulated *S40‐FLAG*, as shown by immunoblot analysis with anti‐FLAG antibodies (Figure 4I). The aphid resistance of *S40*‐OE plants was lower than that of the wild type, as the *S40‐*OE lines had significantly more aphids than the wild type after the initial infestation (Figure 4J). Moreover, the amount of honeydew secreted, the survival rate, and the body weight of aphids were significantly higher when the aphids fed on the leaves of *S40‐*OE plants than on those of the wild type (Figure 4K–O). These results confirm that *S40* acts as a negative regulator of aphid resistance in maize.

### 2.6 *RTA9* Negatively Modulates *S40* Protein Stability

**Figure 5:**
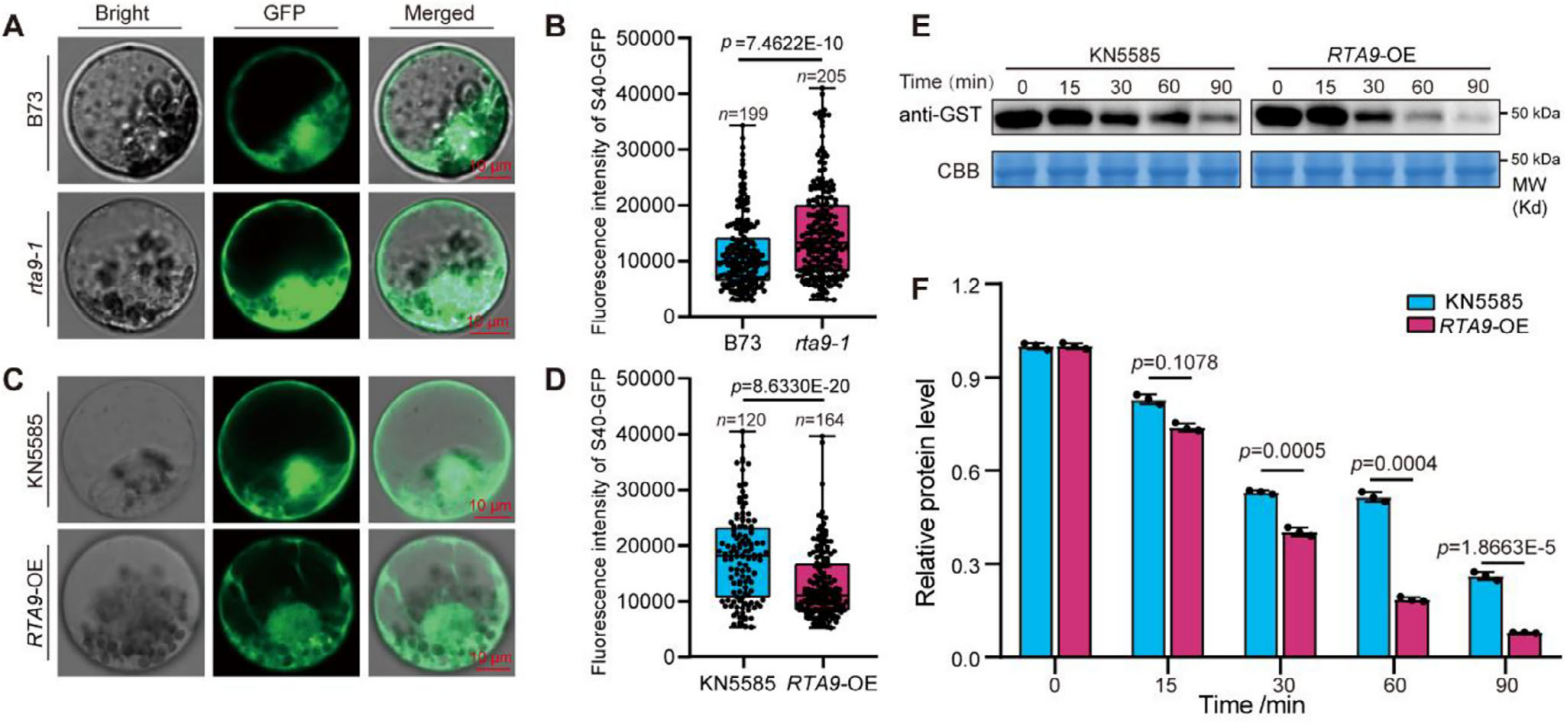
*RTA9* negatively modulates *S40* protein stability in vivo and in vitro. A,B) Fluorescence intensity of *S40‐GFP* in B73 and *rta9‐1* protoplasts (A) and quantification of fluorescence levels (B). C,D) Fluorescence intensity of *S40‐GFP* in KN5585 and *RTA9*‐OE protoplasts (C) and quantification of fluorescence levels (D). E,F) Cell‐free degradation assay showing that the degradation of *S40* is slower in extracts from the wild‐type KN5585 than from those of *RTA9*‐OE. Representative immunoblots are presented in (E) and the relative protein abundance is shown in (F). *S40‐GST* was detected using anti‐GST antibodies, and Coomassie Brilliant Blue (CBB) staining was used to ensure equal loading. In (B, D), the boxes represent the interquartile range, with the middle line defining the median. The lines extending from the quartiles of the box are called “whiskers” and show the maximum and minimum values. In (F), data are shown as means ± SD, and significant differences were determined with Student's *t*‐tests. Experiments in (E, F) were performed at least three times with similar results.

To elucidate the effect of the *RTA9–S40* interaction, we compared the fluorescence intensity of *S40‐GFP* in protoplasts prepared from the wild‐type lines B73 and KN5585, as well as from the *rta9‐1*, and *RTA9‐*OE plants. The *S40‐GFP* fluorescence intensity was significantly higher in protoplasts from the *rta9‐1* mutant than from the wild‐type B73 (**Figure** **5**A,B). However, it was significantly lower in *RTA9‐*OE protoplasts than in those from the control KN5585 (Figure 5C,D), suggesting that *RTA9* negatively influences the accumulation of *S40*.

To further test whether *RTA9* affects *S40* stability, we performed a cell‐free protein degradation assay using recombinant purified *S40‐GST* produced in *Escherichia coli*. We added *S40‐GST* to a protein extract prepared from either the wild‐type KN5585 or *RTA9*‐OE seedlings and collected aliquots over time for analysis by immunoblotting using anti‐His antibodies. We established that recombinant *S40‐GST* is degraded significantly faster in the protein extracts from the *RTA9*‐OE lines than in those from KN5585 (Figure 5E,F). These results suggest that *RTA9* negatively modulates the stability of *S40* protein both in vitro and in vivo.

### 2.7 The Loss Function of *RTA9* Results in an Imbalance of ROS Homeostasis

To determine the mechanism underlying the role of *RTA9* in aphid resistance, we investigated the differentially expressed genes (DEGs) in *rta9‐1* and *s40* relative to their respective wild‐types B73 and B104 using transcriptome deep sequencing (RNA‐seq) analysis. To investigate the role of *RTA9* in the maize response to aphids, we performed pairwise comparisons within each genetic background: aphid‐infested versus noninfested B73 (Tr_B73 versus CK_B73), and aphid‐infested versus. noninfested *rta9‐1* mutant (Tr_*rta9* versus CK_*rta9*). Finally, we defined lists of genotype‐specific DEGs by examining the overlap between the DEGs identified in B73 and *rta9‐1* (Figure S10A, Supporting Information). We thus identified 281 genes as common DEGs in B73 and *rta9‐1*, with an additional 3193 genes specifically expressed in B73, and only 521 genes uniquely induced in the *rta9‐1* mutant (Figure S10A, Supporting Information). A Gene Ontology (GO) term enrichment analysis revealed that over 200 DEGs following aphid infestation of B73 are related to oxidoreductase activity, whereas the equivalent genes were not differentially expressed in *rta9‐1* (Figure S10B,C, Supporting Information). This finding indicates that aphids can induce changes in the expression of genes related to oxidoreductase activity in maize in an *RTA9‐dependent* manner.

**Figure 6:**
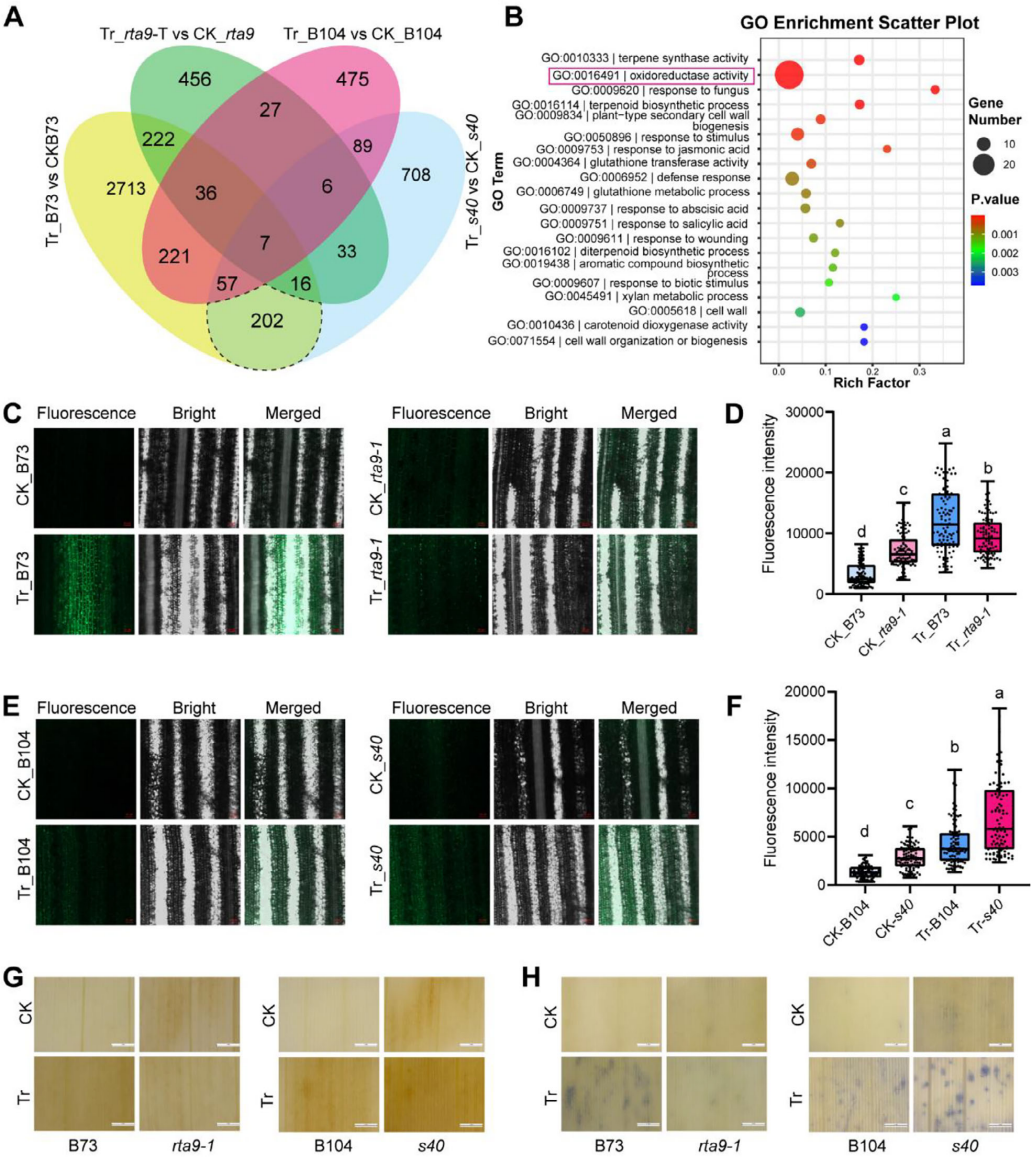
*RTA9* and *S40* regulate the cellular redox status. A) Venn diagram showing the extent of overlap between the number of differentially expressed genes (DEGs) in the wild‐type B73, *rta9‐1*, B104, and *s40* (control, CK; Tr, aphid infestation). The set of genes highlighted by the dashed line represents those that exhibit common changes in B73 and *s40* in response to aphid infestation. B) Gene Ontology (GO) term enrichment analysis of DEGs in the region highlighted by the gray dashed line in (A). C–F) Imaging of ROS levels, detected with H_2_DCFDA, in B73 and *rta9* (C) and in B104 and *s40* (E) under control conditions (CK) and upon aphid infestation (Tr). Quantification of the fluorescence intensity in (C) and (E) is shown in (D) and (F), respectively. G) 3,3′‐diaminobenzidine (DAB) histochemical staining of leaves from B73, *rta9‐1*, B104, and *s40* plants under control conditions or in response to aphid infestation. H) Nitroblue tetrazolium (NBT) histochemical staining of leaves from B73, *rta9‐1*, B104, and *s40* plants under control conditions or in response to aphid infestation. In panels (D, F), at least six individual leaves were stained and photographed. Fluorescence intensity was quantified from 100 randomly selected regions (*n* = 100). The boxes represent the interquartile range, with the middle line defining the median. The lines extending from the quartiles of the box are called “whiskers” and show the maximum and minimum values. Significant differences were determined using one‐way ANOVA followed by Tukey's test (D and F) (*p* < 0.05), with different letters indicating significant differences.

Additionally, 158 DEGs were shared between B104 and *s40*, while 759 DEGs were specific to aphid‐infested B104 and another 959 DEGs were specific to aphid‐infested *s40* (Figure S10D, Supporting Information). GO term enrichment analysis revealed that the pathway related to oxidoreductase activity ranked eighth (*Q*‐value, 0.0014) in the wild‐type B104 after aphid infestation, while the pathway rose to first (*Q*‐value, 3.4328E‐09) in the more aphid‐resistant *s40* (Figure S10E,F, Supporting Information), suggesting that the functional loss of *S40* alters the expression of oxidoreductase activity‐related genes in response to aphids. The DEGs shared by aphid‐infested B73 and aphid‐infested *s40* (highlighted by the gray dashed line in **Figure** **6**A) were enriched in several reported insect resistance pathways, including terpene biosynthesis, cell wall biosynthesis, JA/SA response, and oxidoreductase activity (Figure 6A,B). This result suggests that *RTA9* and *S40* contribute to maize aphid resistance through multiple mechanisms.

The observation that the oxidoreductase activity pathway was enriched in DEGs prompted us to investigate ROS accumulation in *rta9‐1* and *s40*, along with their respective wild types. Accordingly, we stained the leaves with the ROS probe H_2_DCFDA and quantified the resulting fluorescence intensity of its oxidized products. We determined that noninfested *rta9‐1* plants accumulate significantly higher levels of ROS than the wild type B73 (Figure 6C,D). However, following aphid infestation, ROS levels rose significantly in B73 beyond those seen in *rta9‐1*. Aphid infestation also induced the accumulation of ROS in *rta9‐1* to levels above those measured in noninfested plants, but to a lesser extent than observed in B73, resulting in significantly lower ROS accumulation than in aphid‐infested B73 (Figure 6C,D). This result was also supported by 3,3′‐diaminobenzidine (DAB) and nitroblue tetrazolium (NBT) staining (Figure 6G,H). These findings suggest that aphid infestation alters the oxidative balance in maize cells, which is dependent on *RTA9*. By contrast, ROS content exhibited an opposite pattern in *s40*, showing significantly higher levels in *s40* compared to B104 regardless of aphid infestation (Figure 6E–K). We also examined ROS levels in *RTA9*‐OE and *S40*‐OE plants, as well as their respective wild‐types KN5585 and B104. Based on fluorescence intensity, the ROS content was significantly higher in *RTA9*‐OE lines than in the wild type (Figure S11A,B,E,F, Supporting Information), whereas *S40*‐OE cells had significantly lower levels of ROS than the wild type (Figure S11C–F, Supporting Information). These results suggest that the *RTA9–S40* interaction may regulate aphid resistance by altering ROS homeostasis in maize.

## 3 Discussion

In the ongoing evolutionary arms race between plants and their insect pests, plants have evolved a diverse array of defense strategies^[^ ^4^, ^43^ ^]^ that include the precise regulation of anti‐insect gene expression to protect against infestation, thereby enhancing their adaptive and resistance capabilities.^[^ ^1^, ^44^ ^]^ However, the molecular mechanisms governing a substantial portion of these defense‐related genes remain poorly understood. In this study, we demonstrate that the mitochondrial protein *RTA9* and its interaction partner *S40* modulate ROS homeostasis to enhance resistance to aphids in maize.

During infestation, aphids first use their stylets to probe the plant epidermis before penetrating the phloem and subsequently secreting saliva into the mesophyll cells of the host. This action, along with stimuli such as physical damage and effector protein injection, triggers the production of various types of ROS.^[^ ^22^, ^45^, ^46^ ^]^ Here, our transcriptome analysis showed that a substantial number of differentially expressed genes following aphid feeding were enriched in genes related to oxidoreductase activity in B73, but not in *rta9‐1* (Figure S10A–C, Supporting Information). In agreement with this observation, ROS levels were elevated in the leaves of B73 and *rta9‐1* plants following aphid feeding relative to their non‐infested controls, although they reached significantly lower levels in infested *rta9‐1* than in infested wild‐type plants (Figure 6C,D,G,H). The *RTA9*‐OE lines accumulated higher ROS levels than their wild‐type KN5585 (Figure S11, Supporting Information), which aligns with the positive regulatory role of *RTA9* in aphid resistance (Figures 1 and 2). Previous studies showed that the production ROS (H_2_O_2_) in plants negatively affected aphid growth;^[^ ^22^, ^23^, ^47^ ^]^ thus, the lower ROS levels in *rta9* plants may explain why aphids prefer to colonize these plants over the wild type. Additionally, we noticed that pathways related to terpene biosynthesis, cell wall biosynthesis, response to JA, and response to SA were enriched among the DEGs following aphid infestation. This observation suggests that *RTA9* and *S40* may contribute to the resistance of maize plants to aphids in multiple ways.

Mitochondria are semiautonomous organelles with various ETCs and are a major source of ROS in cells.^[^ ^48^ ^]^ Changes in mitochondrion‐localized proteins tend to alter the balance of intracellular ROS, which in turn affects plant resistance.^[^ ^1^, ^21^, ^49^ ^]^ For instance, the mitochondrial protein RTP7 modulates ROS levels by affecting the subunits of mitochondrial respiratory chain complex I, whereas the loss of RTP7 function enhances plant resistance to a broad spectrum of plant pathogens and salinity stress.^[^ ^34^ ^]^ Similarly, a mutant lacking the mitochondrion‐localized protein NATURAL BLIGHT LEAF 3 in rice accumulates H_2_O_2_ and exhibits enhanced resistance to biotic and abiotic stresses.^[^ ^49^ ^]^ Considering the subcellular localization of *RTA9* (Figure 3A), we hypothesize that it may be involved in mitochondrial functions, such as the respiratory chain. The loss of *RTA9* function likely disrupts mitochondrial electron transport, resulting in higher baseline ROS levels in the *rta9‐1* mutant compared to the wild type (Figure 6C,D,G,H). Consequently, *rta9* mutants accumulated ROS at a constitutively higher baseline level than the wild type (Figure 6C–H). However, upon aphid infestation, the *rta9‐1* mutant showed a more modest increase in ROS levels compared to the wild‐type control (Figure 6C–H). Conversely, *RTA9*‐OE lines displayed elevated ROS levels and enhanced aphid resistance (Figure 2H–M; Figure S11, Supporting Information). These results demonstrate that the mitochondrial protein *RTA9* is required for ROS accumulation during aphid infestation. Thus, impaired RTA9‐mediated ROS accumulation in the *rta9* mutants limits oxidative bursts, ultimately leading to increased susceptibility to aphids.

Protein subcellular localization, LUC complementation imaging assays, BiFC assays, Co‐IP assays, and pull‐down analysis all indicated that *RTA9* and *S40* partially co‐localize and physically interact in vitro and in vivo (Figure 3). Furthermore, *RTA9* negatively regulates the stability of S40 (Figure 5), which explains why these proteins affect aphid resistance in contrasting ways. For instance, *rta9* mutants are more susceptible to aphid infestation than the wild‐type B73 (Figure 1), whereas the *s40* mutant is more resistant than its wild‐type B104 (Figure 4). *S40* is a homolog of the DUF584 family member HvS40 in barley (*Hordeum vulgare*), which was first reported to be associated with leaf senescence.^[^ ^50^ ^]^ *HvS40* expression is upregulated under various senescence conditions, from dark‐induced senescence to age‐dependent senescence in barley leaves, making it a useful marker gene for senescence.^[^ ^51^ ^]^ To our knowledge, DUF584 has not been reported to be associated with insect resistance in plants. Here, we report that the overexpression or knockout of *S40* can result in variations in ROS levels (Figure 6E–H; Figure S11, Supporting Information), which are associated with changes in aphid resistance (Figure 4). Previous studies have shown that cytoplasmic signaling regulates nuclear gene expression through retrograde signaling.^[^ ^33^ ^]^ *RTA9* is localized to mitochondria and regulates *S40* stability, while *S40* is evenly distributed within plant cells, including the mitochondria and the nucleus, and its loss of function alters ROS homeostasis. It is therefore possible that the regulation of the *RTA9–S40* module may also be achieved through retrograde signaling, a possibility that merits further investigation. The mechanisms by which *RTA9* influences *S40* stability should also be further explored.

Based on the above results, we propose that *RTA9* mediates aphid‐triggered signaling in maize, responding to physical damage or the saliva proteins of aphids, thereby activating the signaling pathway dependent on a ROS burst. In summary, the mitochondrion‐localized *RTA9–S40* protein complex is involved in regulating aphid resistance in maize, likely by modulating the plant's redox status. *RTA9* overexpression and knockout of *S40* confer aphid resistance but do not reduce maize seed production in the field, suggesting that *RTA9* and *S40* are potential candidate genes for breeding aphid‐resistant maize varieties.

## 4 Experimental Section

### Plant Growth and Aphid Culture Conditions

Maize (*Zea mays*) and *Nicotiana benthamiana* plants were grown in a greenhouse under a 16‐h light/8‐h dark photoperiod and 28 °C/22 °C (day/night) cycles. Aphids (*Rhopalosiphum maidis*) were collected from maize fields in Hefei, China, and artificially bred in greenhouses for over 10 generations before use. All seedlings used for the aphid assays were cultured under a 59 × 61 × 91 cm nylon net cage to ensure there was no disturbance from other pests.

An M_2_ population from an EMS‐mutagenized library in the B73 background was planted in Hainan (18°21′N, 109°10′E), China.^[^ ^3^, ^40^ ^]^ Aphid‐susceptible and aphid‐resistant plants were scored, self‐pollinated, and harvested for study. Among them, the aphid‐susceptible mutant *rta9‐1* was identified. The Mu‐insertion mutant *rta9‐2* was ordered from the ChinaMu database (http://chinamu.jaas.ac.cn/Default.aspx).^[^ ^41^ ^]^ The single guide RNA (sgRNA) used for CRISPR/Cas9‐mediated gene editing was designed using CRISPR‐GE (http://skl.scau.edu.cn/home/),^[^ ^52^ ^]^ and transgenic maize lines were generated by Beijing BomeiXingao Technology (Beijing, China). Mutant plants were selected for backcrossing to the wild‐type B73, and were self‐crossed again to eliminate any other potential second‐site mutations. Homozygous mutant seeds were collected and used for the experiments.

### Gene Cloning using the MutMap Method

The MutMap method^[^ ^40^, ^53^ ^]^ was used to clone the candidate gene underlying the aphid‐susceptible phenotype of *rta9‐1*. The aphid‐sensitive mutant was backcrossed to the parental inbred line B73, and the resulting F_1_ plants were self‐crossed. The F_2_ progeny was analyzed for aphid accumulation and aphid‐susceptible individuals were identified. The leaves of 25 F_2_ seedlings exhibiting the aphid‐susceptible phenotype were collected. Genomic DNA was extracted from individual seedlings using the cetyltrimethylammonium bromide (CTAB) method, its concentration quantified, and mixed in an equimolar ratio for each sample to create one pool. The resulting DNA pool was subjected to genome resequencing by Biomarker Technologies (Beijing, China). After the candidate gene was cloned and the target mutation sites were revealed, the derived cleaved amplified polymorphic sequence (dCAPS) method was used to validate the mutation. The primer sequences are listed in Table S1 (Supporting Information).

### RNA Extraction, cDNA Synthesis, and RT‐qPCR

Total RNA was extracted from samples using Trizol reagent (Takara Bio, Dalian, China) and the first‐strand cDNA was synthesized with a PrimeScript RT Reagent Kit (Takara Bio) from 1 µg of total RNA following the manufacturer's protocol. For tissue‐specific expression analysis, silk and tassel samples were collected at the flowering stage, while all other tissues were collected from maize plants at the four‐leaf stage. Gene expression analysis in transgenic maize was conducted using the fourth leaf harvested from seedlings at the four‐leaf stage. qPCR was performed on a Roche LC480 LightCycler (Roche, Basel, Switzerland) using gene‐specific primers. The relative expression level of each gene was normalized to that of *GAPD*H by the 2^−ΔΔ^ Ct method.^[^ ^54^ ^]^ Each experiment was conducted with three biological replicates and two technical replicates. The sequences of all primers are included in Table S1 (Supporting Information).

### Aphid Survival Rate and Honeydew Excretion

For the test of aphid survival rates, previously published methods were followed.^[^ ^3^ ^]^ To ensure that no aphids reproduced during the experiment, 20 one‐day‐old aphids were gently placed onto the second leaf of seedlings at the four‐leaf stage. The number of aphids on each seedling was determined over seven consecutive days, and the aphids were also weighed on the seventh day. Five aphids were weighed together to calculate the weight per aphid.

The amount of aphid honeydew excretion is a direct indicator of aphid feeding. For the honeydew excretion test, seedlings were cultivated with the same number of aphids as described above. After two days, a piece of perforated filter paper was placed horizontally on the seedling stalks. Two days later, the filter papers were collected, sprayed with 0.1% (w/v) ninhydrin acetone solution, and dried in an oven at 60 °C for 30 min. Finally, the color‐developed filter papers were scanned and the spot areas were quantified using ImageJ software (v. 1.54f, National Institutes of Health, Bethesda, MD, USA). Each biological experiment was independently repeated at least three times, with six leaves included per replicate.

### Subcellular Localization and Co‐Localization Analysis

To investigate the subcellular localization of *RTA9* and *S40*, their full‐length coding sequences without the stop codon were cloned individually into a modified pCAMBIA1305 vector containing a green fluorescent protein (*GFP*) sequence and the cauliflower mosaic virus (CaMV) 35S promoter.^[^ ^55^ ^]^ The *S40* coding sequence was also cloned in‐frame with the sequence of the red fluorescent protein (*RFP*) in pCAMBIA1300. Maize protoplasts were isolated through enzymatic digestion and the plasmids were transferred into the protoplasts using the polyethylene glycol (PEG) method.^[^ ^54^ ^]^ After incubation for 24 h in the dark, GFP and RFP fluorescence signals were captured using a laser confocal microscope (LSM800; Carl Zeiss, Oberkochen, Germany).

### Yeast Two‐Hybrid Assay (Y2H)

The full‐length coding sequences of *RTA9* and *S40* were cloned into the pGAD‐T7 and pGBK‐T7 vectors, respectively. The resulting plasmids were introduced into the yeast (*Saccharomyces cerevisiae*) strain AH109. Positive transformants were selected for growth on synthetic defined (SD) medium lacking Trp and Leu at 30 °C for three days. Positive colonies were resuspended in sterile ddH_2_O and washed with sterile ddH_2_O at least three times, after which the cells were spotted onto SD medium lacking Leu, Trp, His, and Ade and cultured at 30 °C for three days.

### Firefly LUC Complementation Imaging Assay

For the LUC complementation assays, the full‐length coding sequences of *RTA9* and *S40* were cloned into the appropriate cloning sites of the *nLUC* or *cLUC* vector, respectively, using restriction enzyme digestion and ligation methods.^[^ ^56^ ^]^ The resulting plasmids were introduced individually into Agrobacterium (*Agrobacterium tumefaciens*) strain GV3101 containing pSoup + p19. Pairs of Agrobacterium cultures harboring each plasmid were resuspended in infiltration buffer (0.01 M MES, 0.01 mM MgCl_2_, 0.1 mM acetosyringone) and mixed in a 1:1 ratio before being co‐infiltrated into the leaves of *N. benthamiana* plants. After three days of culture in an incubator, the leaves were uniformly sprayed with 1 mM D‐luciferin, and the LUC signal was captured with a chemiluminescence camera (5200; Tanon Science and Technology, Shanghai, China).

### Bimolecular Fluorescence Complementation (BiFC) Assay

For the BiFC assays, the full‐length coding sequences of *RTA9* and *S40* were cloned into pCAMBIA1300‐YCE or pCAMBIA1300‐YNE vectors, respectively, at appropriate cloning sites via restriction digestion. The resulting plasmids were transfected into maize protoplasts using the PEG method. The transfected cells were cultured for 24 h at 26 °C in the dark, after which YFP signals were observed by confocal fluorescence microscopy (LSM800; Carl Zeiss, Oberkochen, Germany).

### Recombinant Protein Production and In Vitro Protein Pull‐Down Assays

For the in vitro protein pull‐down assays, RTA9‐MBP‐His and *S40‐GST* were produced in *E. coli*.^[^ ^56^ ^]^ Briefly, the full‐length coding sequence of *RTA9* was inserted into a modified pET28a(+) vector containing the *MBP* sequence; the full‐length coding sequence of *S40* was cloned into a pGEX‐6P‐1 vector containing the *GST* sequence. The resulting plasmids were introduced into *E. coli* BL21(DE3) and the production of each recombinant protein was induced by incubation at 16 °C for 24 h in the presence of 0.1 mM isopropyl‐β‐D‐thiogalactopyranoside (IPTG). The cell pellets were collected by centrifugation at 4 °C, and then lysed by sonication (XM‐650T, Jingxin, China). The proteins in the supernatant were collected by centrifugation and purified with Ni^2+^‐NTA and glutathione agarose beads (Solarbio, Beijing, China). RTA9‐MBP‐His or the negative control MBP‐His was mixed with *S40‐GST*, and the mixtures were then added to 5 µL of His trap beads (ChromoTek, Planegg, Germany) and incubated at 4 °C for at least 4 h. The input and pulled‐down proteins were detected using anti‐His (Roche) or anti‐GST antibodies (EnoGene), respectively.

### In Vivo Co‐Immunoprecipitation (Co‐IP) Assays

For in vivo Co‐IP assays, the *RTA9‐GFP* transgenic line was crossed to the *S40‐FLAG* transgenic line to obtain F_1_ *RTA9‐GFP S40‐FLAG* plants. Then, 25 g of these F_1_ seedlings at the four‐leaf stage were collected and ground into fine powder in liquid nitrogen and then resuspended in 25 mL of extraction buffer (50 mm Tris‐HCl, pH 8.0; 150 mm NaCl; 0.1% [v/v] IGEPAL; 2.5 mm EDTA, 10% [v/v] glycerol; 10 mm β‐mercaptoethanol; 1 mM PMSF; 10 µm leupeptin; and 1× Roche protease inhibitor cocktail [Roche, Basel, Switzerland]).^[^ ^56^ ^]^ The resulting mixture was incubated on ice for 10 min and then centrifuged at 12000 × *g* for 20 min at 4 °C. The supernatant was further clarified by repeating the centrifugation until it was clear. GFP Trap beads, pre‐washed with extraction buffer, were then added to the protein extracts and incubated at 4 °C for 3 h. Following three washes with extraction buffer, the beads were collected, boiled in SDS loading buffer, and subjected to SDS‐PAGE for immunoblot analysis. Target proteins were detected using anti‐GFP (Roche, Basel, Switzerland) or anti‐FLAG (MBL, Sapporo, Hokkaido, Japan) antibodies.

### In Vitro Protein Degradation Assays

Recombinant purified S40‐GST was incubated with total protein extracts from B73 or *rta9‐1* to assess its stability. Briefly, maize leaves were ground to a powder in liquid nitrogen and their total proteins were extracted by adding 1 mL protein extraction buffer (50 mm Tris‐HCl, pH 7.5, 150 mm NaCl, 0.1% [v/v] IGEPAL, 2.5 mm EDTA, 10% [v/v] glycerol, 10 mm 2‐mercaptoethanol, 10 µm leupeptin) to each 1 g of powder. The proteins in the supernatant (soluble proteins) were obtained by centrifugation at 12000 ×*g* for 20 min at 4 °C, after which ≈ 20 µg of recombinant *S40‐GST* was added to 1 mL of maize protein extract and incubated at 28 °C for different durations. *S40‐GST* abundance was detected using an anti‐GST antibody (Abmart, Shanghai, China) after SDS‐PAGE and transfer to PVDF membrane.

### Transcriptome Analysis

For RNA‐seq analysis, maize seedlings were grown to the four‐leaf stage in the greenhouse before 20 adult aphids were moved onto their second leaves for 24 h. The leaves and sheaths from six independent maize seedlings were collected as one experimental replicate. Total RNA was extracted from the samples using an RNeasy kit (Thermo Fisher Scientific, Waltham, MA, USA). Three independent experimental replicates were performed for each sample. The control group (CK) consisted of plants not infested with aphids, defined as CK_B73, CK_*rta9*, CK_B104, and CK_*s40*. The treatment group (Tr) comprised aphid‐infested plants, designated as Tr_B73, Tr_*rta9*, Tr_B104, and Tr_*s40*.

cDNA libraries were sequenced using a NovaSeq 6000 instrument (Illumina, San Diego, California, USA) by Lianchuan Biotechnology Company (Hangzhou, China). The sequencing and analysis methods were described previously.^[^ ^10^ ^]^ The fragments per kilobase of exon per million reads mapped (FPKM) values were calculated to normalize the expression data, and the R package DESeq2 was used to determine differential expression among samples.

Clean reads were mapped to the maize B73_v5 reference genome. Differentially expressed genes (DEGs) were defined as those with an absolute fold‐change ≥1.5 with *q* < 0.05. The identified DEGs were subjected to GO term enrichment analysis using the Lianchuan cloud tools (https://www.omicstudio.cn/tool).

### Determination of ROS, H_2_O_2_, and Superoxide Anion (O_2_^−^) Contents

To assess the effect of *RTA9* and *S40* on ROS, the accumulation of ROS, H_2_O_2_, and O_2_^−^ was quantified following the methods described in previous studies.^[^ ^57^, ^58^ ^]^ Briefly, leaves were soaked in staining buffer (10 mm Tris‐HCl, 50 mm KCl, 50 µm H_2_DCFDA, 0.02% [v/v] Tween‐20, pH 7.2) and subjected to vacuum infiltration at −0.6 MPa for 20 min. Excess dye was removed by rinsing the leaves with distilled water, and fluorescence was captured and quantified using a laser confocal microscope (LSM800; Carl Zeiss, Oberkochen, Germany). H_2_O_2_ was detected using 3,3′‐diaminobenzidine staining (DAB, Sigma–Aldrich, St. Louis, MO, USA). Leaf segments were treated with DAB solution (1 mg mL^−1^) for 10 h, followed by decolorization in 80% (v/v) ethanol, which was refreshed until it turned colorless; the samples were observed under a microscope (Leica M165, Germany). O_2_^−^ was visualized through staining with nitroblue tetrazolium (NBT, Servicebio, Wuhan, China). The leaf pieces were incubated in 20 mL of NBT solution (50 mg dissolved in Tris‐HCl buffer pH 7.4) for 10 h, decolorized in 80% (v/v) ethanol, and then visualized under a microscope (Leica M165, Germany).

### Assessment of Agronomic and Yield Traits

To comprehensively evaluate the agronomic and yield traits of the *RTA9*‐OE and *s40*, plants were grown at a density of 25 × 60 cm.^[^ ^59^ ^]^ A randomized block design was employed with two replicates per line. In each replicate, data were collected from two randomly selected rows, with at least 15 individual plants per genotype, excluding the first and last plants in each row. Agronomic and yield traits were assessed as follows: Plant height was measured as the distance from the soil surface to the tip of the tassel at anthesis. Ear height was measured as the distance from the stem base to the node bearing the uppermost ear. Ear leaf length was recorded as the distance from the tip of the ear leaf to the top of its sheath. Ear leaf width was recorded at the maximum width of the ear leaf midpoint. Ear length was measured as the total length of the mature ear, including both filled and unfilled portions. Ear diameter was measured at the widest point of the mature ear. 100‐kernel weight was determined by weighing 100 randomly selected kernels after drying. Ear weight was determined as the dry weight of the ear, including cob and kernels. Grain yield per plant was determined as the dry weight of all kernels per plant after threshing. For each trait, at least 15 independent plants were assessed, except for 100‐kernel weight, which was measured with six independent replicates.

### Statistical Analysis

All data obtained in this study were analyzed using SPSS Statistics 20.0 software (IBM, Armonk, New York, USA) and GraphPad Prism 8.0.1 (San Diego, CA, USA). For aphid bioassays (population counts, body weight, survival rate, and honeydew excretion), at least six independent biological replicates were performed per experiment. Data are presented as means ± standard deviation (SD), except for body weight (box plots). For RT‐qPCR, experiments were independently performed three times with results expressed as means ± SD. For ROS detection, at least six individual leaves per treatment were stained and photographed, with fluorescence intensity quantified in 100 randomly selected regions. Statistical significance was determined using two‐tailed unpaired Student's *t*‐tests or one‐way analysis of variance (ANOVA) followed by Tukey's test, with *p* < 0.05 considered significant.

## Conflict of Interest

The authors declare no conflict of interest.

## Author Contributions

C.H.W. and X.Q.Z. contributed equally to this work. P.J.L. conceived and supervised the project. P.J.L. and C.H.W. designed the experiments and analyzed the data. C.H.W., X.Q.Z., L.W., S.J.H., J.M., T.Y.W., Y.B.Z., J.H.D., C.C., Z.T., X.Z.C., and N.L. performed the research. C.H.W. wrote the manuscript.

## Supporting information



Supporting Information

## Data Availability

The data that support the findings of this study are available from the corresponding author upon reasonable request.
