# Supplementary material for: The Mitochondrial Protein RESISTANCE to APHIDS 9 Interacts with S40 to Resist Aphid Infestation by Modulating Reactive Oxygen Species Homeostasis in Maize (Zea mays)
Source: Adv Sci (Weinh). 2025 Sep 24;12(46):e04382. doi: 10.1002/advs.202504382 (PMC12697904; doi:10.1002/advs.202504382)
Supplement: Supplementary file 1 — Supporting Information [file ADVS-12-e04382-s001.pdf]

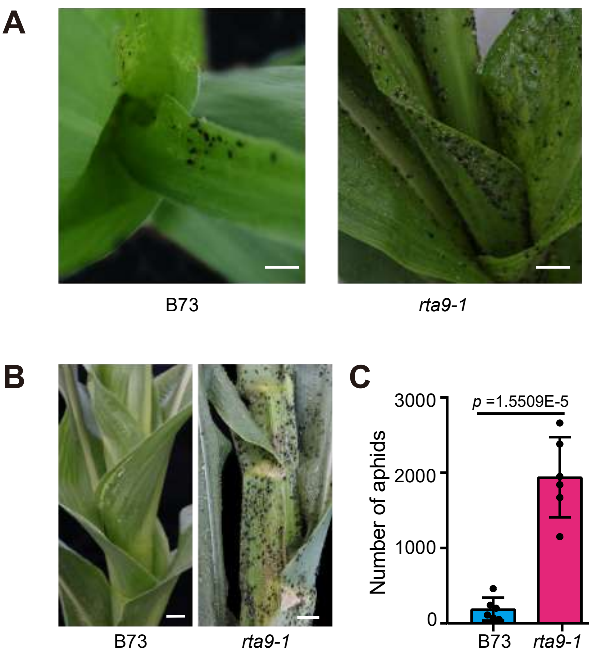

Figure S1. Phenotype of the aphid-susceptible mutant *rta9-1* in the field after artificial infestation. (A, B) Distribution of aphids on different parts of the plant. Scale bar, 1 cm. (C) The number of aphids on plants of the wild-type B73 or the *rta9-1* mutant. The data are shown as means ± SD, n = 6. Statistical significance was determined using Student's t-tests.

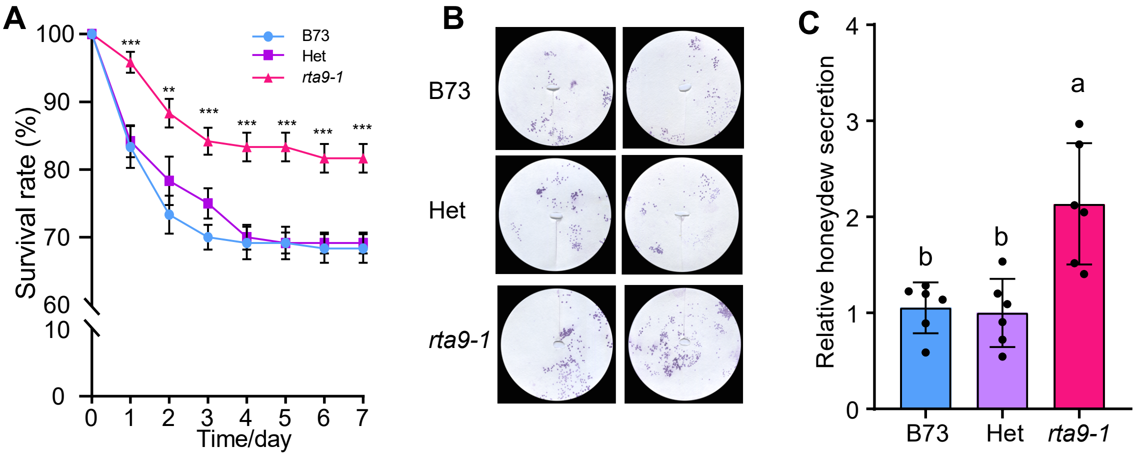

Figure S2. *rta9-1* is a recessive mutant. (A) Survival rate of aphids feeding on B73, seedlings heterozygous for *rta9-1* (Het; *rta9-1/RTA9*), or *rta9-1* seedlings (** $p < 0.01$; *** $p < 0.001$). (B, C) Honeydew secretion assay. Quantification of the honeydew areas on the filter paper in (B) is shown in (C). The data are shown as means ± SD, n = 6. In (A, C), statistical significance was determined using one-way ANOVA followed by Tukey's test ($p < 0.05$), with different letters in (C) indicating significant differences.

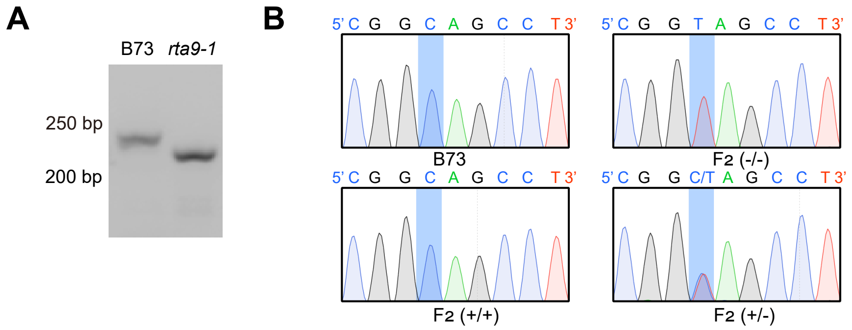

Figure S3. Confirmation of the mutation site in *rta9-1* using a derived cleaved amplified polymorphic sequence (dCAPS) marker and PCR sequencing. (A) Agarose gel electrophoresis analysis of B73 and *rta9-1* samples subjected to dCAPS analysis. (B) Sanger sequencing of the PCR products. The aphid-susceptible plant (F_2 −/−) was homozygous for the mutation, while the other plants had the wild-type allele or were heterozygous.

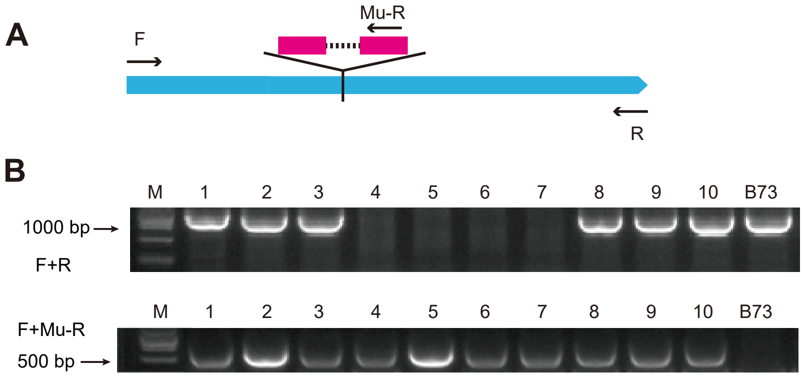

Figure S4. Identification of the *rta9-2* mutant. (A) Diagram of the *RTA9* locus showing the position of the Mu insertion in the *rta9-2* mutant. (B) Identification of the *rta9-2* mutant by PCR genotyping. The primer positions are shown in (A). Lanes 4–7 represent homozygous plants, while other lanes correspond to heterozygous plants; B73 served as a negative control for the presence of the Mu insertion.

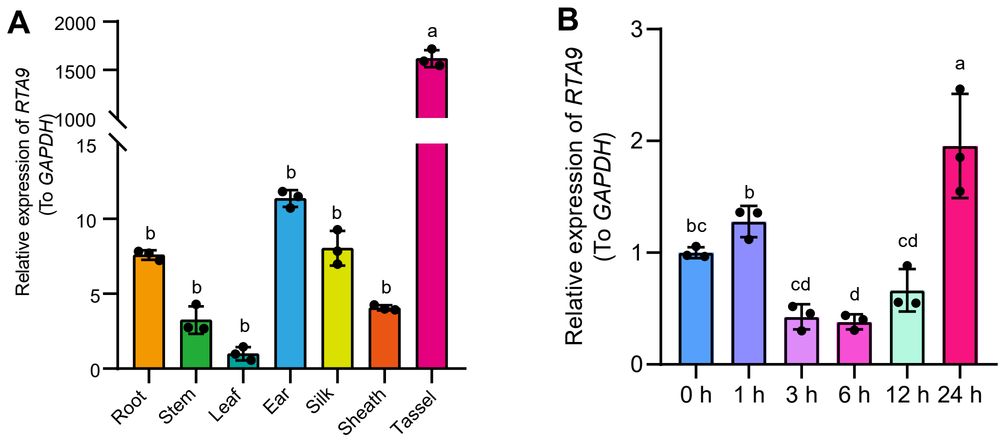

Figure S5. Detection of *RTA9* expression pattern and its transcriptional response to aphid infestation. (A) Expression pattern of *RTA9* in different maize tissues. (B) Time-course analysis of *RTA9* transcript levels following aphid infestation. Data are presented as means ± SD (n = 3). Statistical significance was determined using one-way ANOVA with Tukey's test ($p < 0.05$), with different letters indicating significant differences.

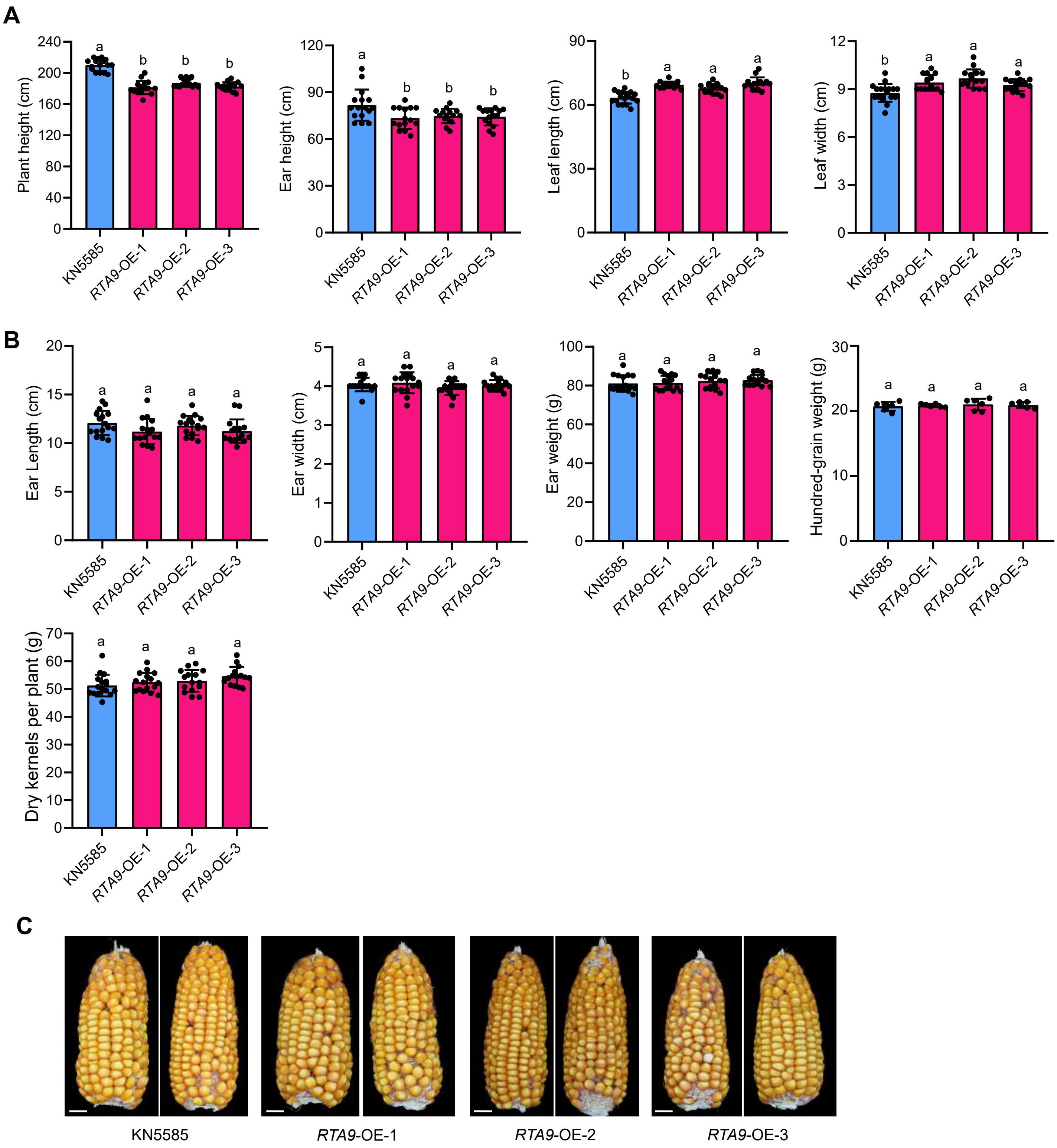

Figure S6. Analysis of agronomic and yield traits of *RTA9*-OE maize lines. (A) Plant architecture traits: plant height, ear height, leaf length, and leaf width. *RTA9*-OE lines exhibited significantly reduced plant and ear heights compared to the wild type, but had longer and wider ear leaves. The data are shown as means ± SD, $n \geq 15$. (B) Yield components: ear length, ear width, ear weight, hundred-kernel weight and weight of dry kernels per plant. No significant differences were observed in ear length, width, or weight ($n \geq 15$) or in hundred-kernel weight ($n = 6$). The data are shown as means ± SD. (C) Representative photographs of ears from the wild-type KN5585 and *RTA9*-OE lines. Scale bar, 1 cm. Statistical significance was determined using one-way ANOVA followed by Tukey's test ($p < 0.05$), with different letters in (A and B) indicating significant differences.

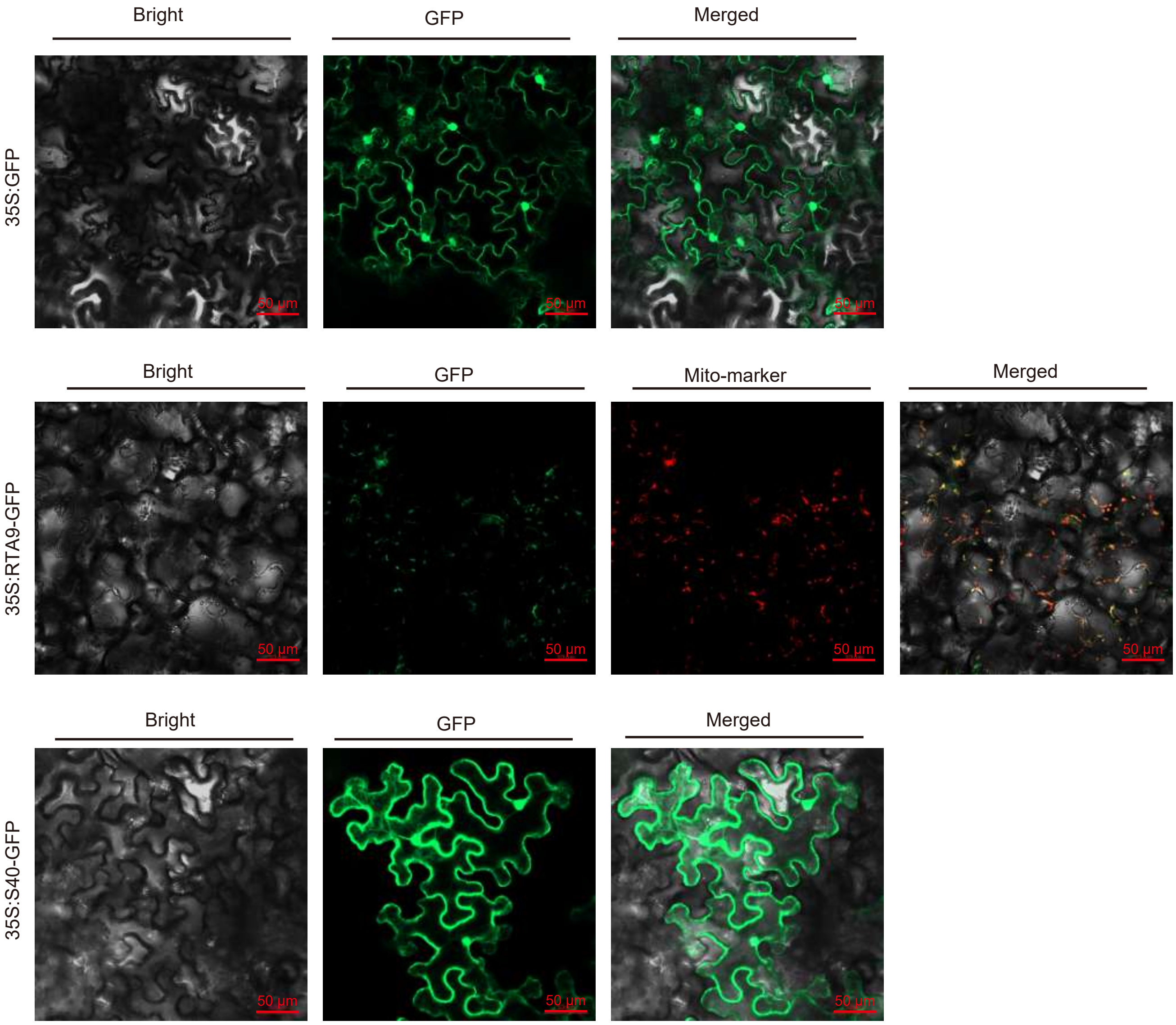

Figure S7. Subcellular localization of RTA9 and S40 in *N. benthamiana* leaf cells. (A) Representative fluorescence images of free GFP derived from the *35S:GFP* construct, used as a positive control. (B) Representative fluorescence images from RTA9-GFP and a mitochondrial marker (CD3-991), showing the overlap of GFP and mCherry signals. (C) Representative fluorescence images of S40-GFP showing its even distribution in the nucleus and cytosol.

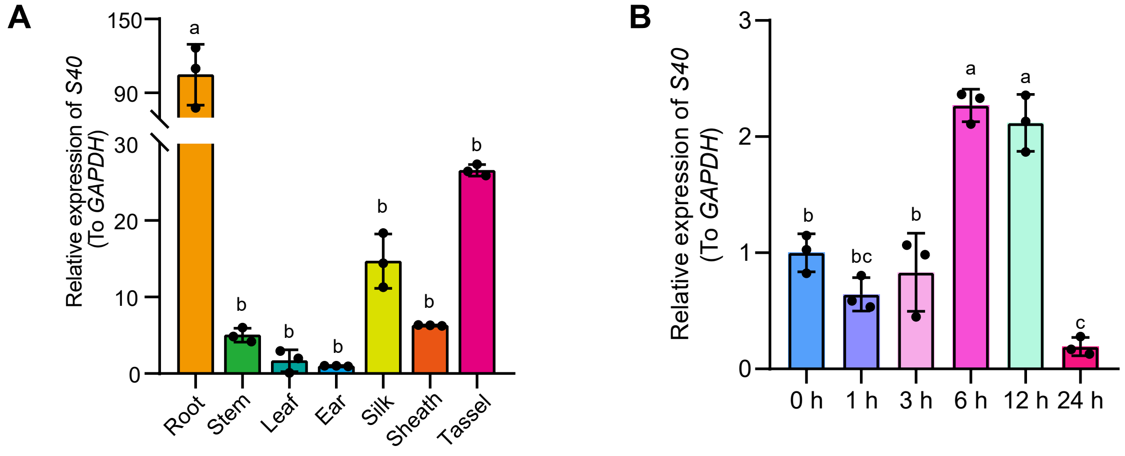

Figure S8. Detection of *S40* expression pattern and its transcriptional response to aphid infestation. (A) Expression pattern of *S40* in different tissues, as determined by RT-qPCR. (B) Time-course analysis of *S40* transcript levels following aphid infestation. Data are presented as means ± SD (n = 3). Statistical significance was determined using one-way ANOVA with Tukey's test (p < 0.05), with different letters in indicating significant differences.

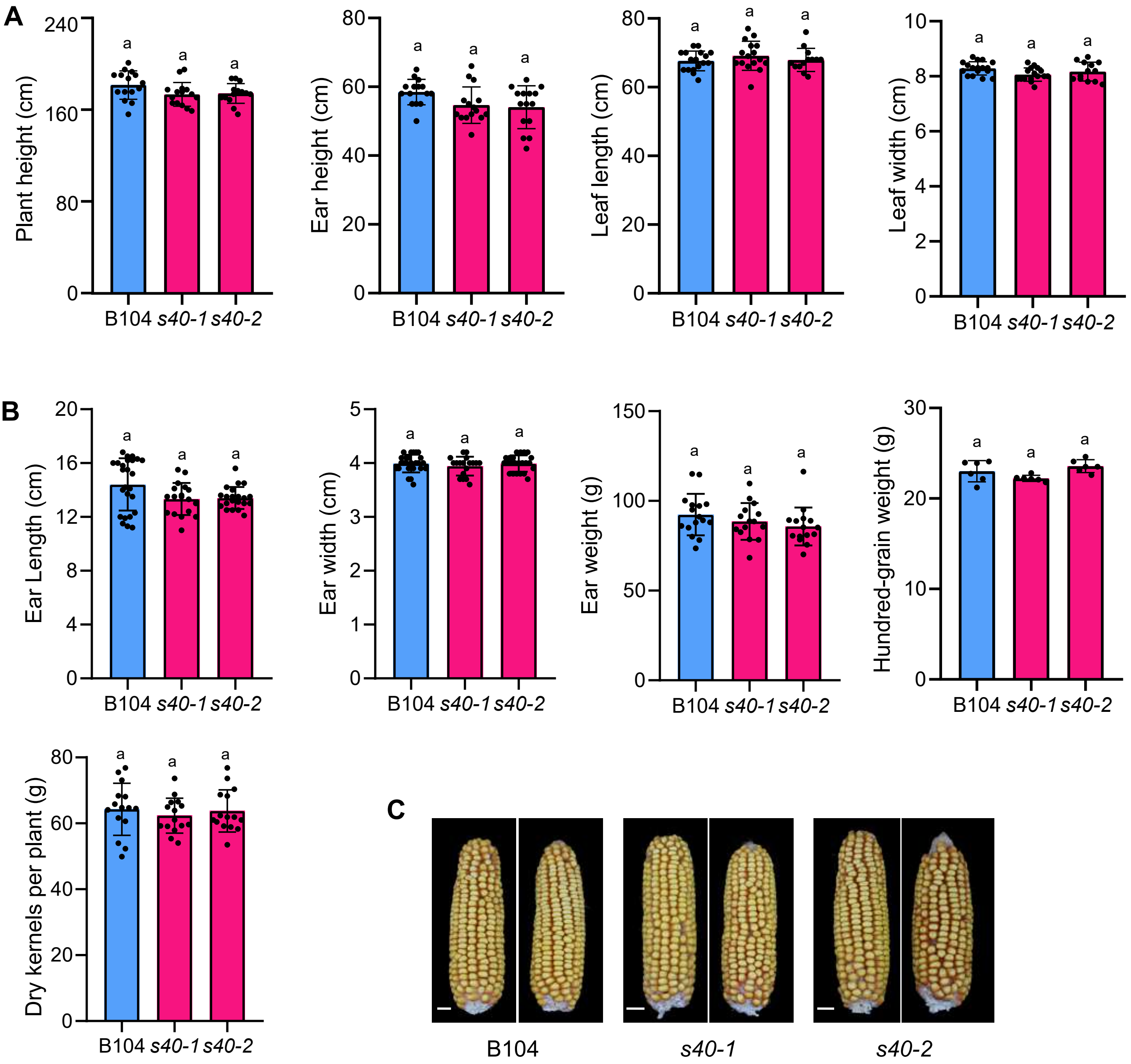

Figure S9. Analysis of agronomic and yield traits of *s40* mutants. (A) Plant architecture traits: plant height, ear height, leaf length, and leaf width. The *s40* mutants showed no significant differences in plant height, ear height, ear-leaf length, or leaf width relative to the wild type. The data are shown as means ± SD ($n \geq 15$). (B) Yield components: ear length, ear width, ear weight, weight of dry kernels per plant, and hundred-kernel weight. No significant differences were observed for ear length, ear width, ear weight ($n \geq 15$), or for hundred-kernel weight (n = 6). The data are shown as means ± SD. (C) Representative photographs of ears from the wild-type B104 and *s40* mutants. Scale bar, 1 cm. Statistical significance was determined by one-way ANOVA with Tukey's test ($p < 0.05$), with different letters in (A and B) indicating significant differences.

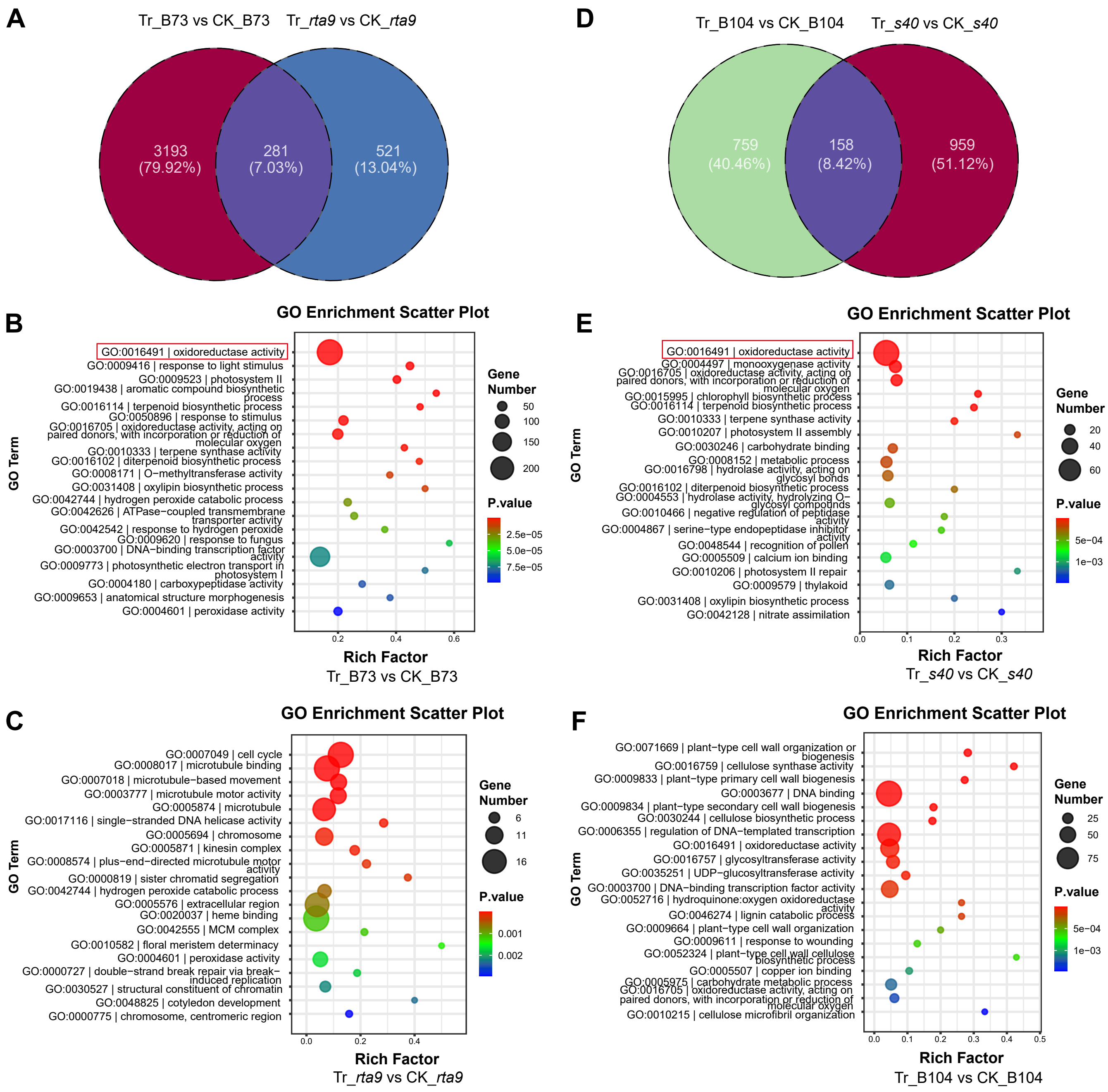

Figure S10. Overview of transcriptome analysis after aphid infestation. (A) Venn diagram showing the extent of overlap between DEGs derived from the pairwise comparisons Tr_B73 vs CK_B73 and Tr_*rta9* vs CK_*rta9*. (B) Top 20 enriched GO terms for the DEGs derived from the Tr_B73 vs CK_B73 comparison. (C) Top 20 enriched GO for the DEGs derived from the Tr_*rta9* vs CK_*rta9* comparison. (D) Venn diagram showing the extent of overlap between DEGs derived from the pairwise comparisons Tr_B104 vs CK_B104 and Tr_*s40* vs CK_*s40*. (E) Top 20 enriched GO terms for the DEGs derived from the Tr_*s40* vs CK_*s40* comparison. (F) Top 20 enriched GO terms for the DEGs derived from the Tr_B104 vs CK_B104 comparison.

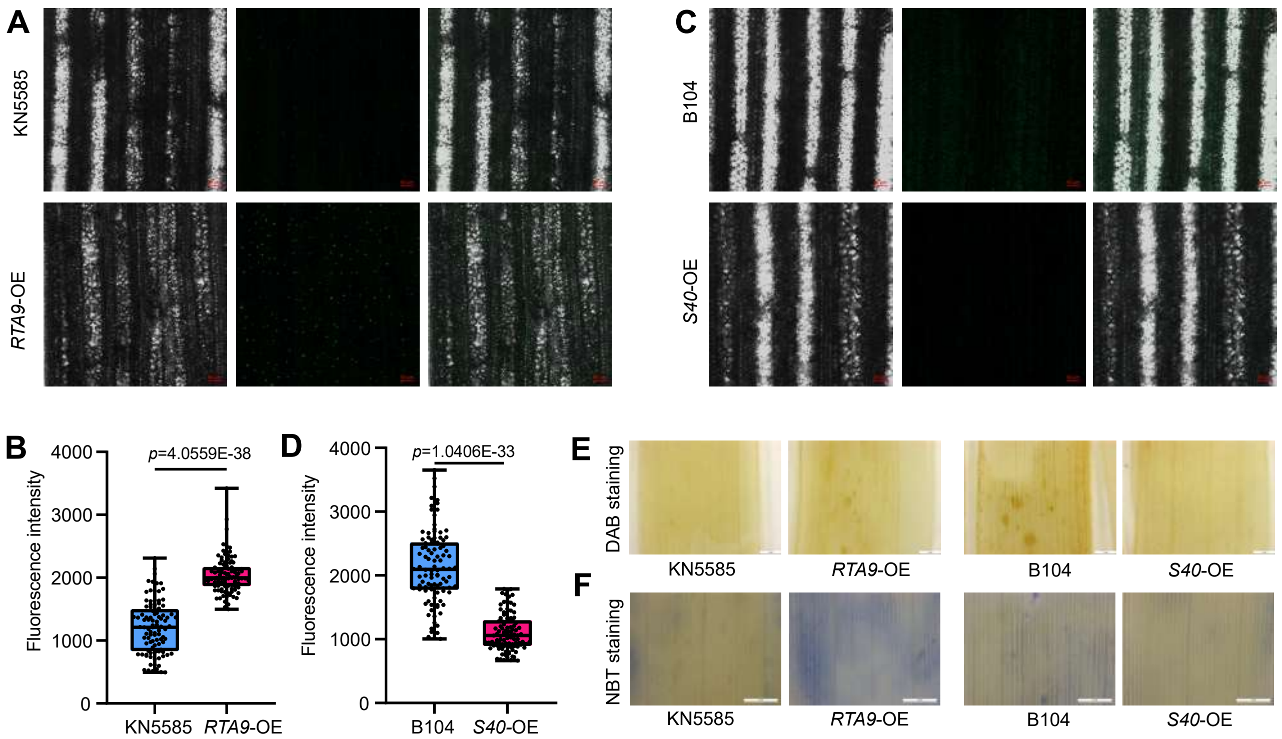

Figure S11. ROS levels in *RTA9*-OE and *S40*-OE leaves. (A) ROS fluorescence intensity in leaves from the wild-type KN5585 and *RTA9*-OE lines, observed after incubating leaves with the ROS probe H_2DCFDA. The quantification of the fluorescence intensity shown in panel (A) is presented in panel (B). (C) ROS fluorescence intensity in the wild-type B104 and *S40*-OE lines, observed after incubation with the ROS probe H_2DCFDA. The quantification of the fluorescence intensity shown in panel (C) is presented in panel (D). (E) and (F) show DAB staining and NBT staining of the KN5585 and *RTA9*-OE and B104 and *S40*-OE lines, respectively. In panels (A) and (C), at least six individual leaves were stained and photographed. Fluorescence intensity was quantified from 100 randomly selected regions. In panels (B) and (D), the boxes represent the interquartile range, with the middle line defining the median. The lines extending from the quartiles of the box are called ‘whiskers’ and show the maximum and minimum values (n = 100). Statistical significance was determined using Student's t-tests.
